# Integrating care between an NHS hospital, a community provider and the role of commissioning: the experience of developing an integrated respiratory service

**DOI:** 10.1136/bmjopen-2020-040267

**Published:** 2020-12-21

**Authors:** Jonathan Banks, Tracey Stone, James Dodd

**Affiliations:** 1 The National Institute for Health Research, Applied Research Collaboration West (NIHR ARC West), University Hospitals Bristol NHS Foundation Trust, Bristol, UK; 2 Population Health Sciences, Bristol Medical School, University of Bristol, Bristol, UK; 3 Academic Respiratory Unit, Translational Health Sciences, University of Bristol, Southmead Hospital, Bristol, UK

**Keywords:** organisation of health services, qualitative research, adult thoracic medicine, health policy

## Abstract

**Objectives:**

An integrated respiratory service was commissioned in 2016 in a UK region to support patients with chronic obstructive pulmonary disease. The service brought together the respiratory department of a National Health Service hospital and a not-for-profit community provider. This paper evaluates: (1) the perceived efficacy of integrated working between the organisations from the perspective of staff and (2) the relationship between commissioning and integration of the services.

**Design:**

Semistructured interviews with staff from the three organisations involved in the integrated respiratory service. Staff were purposefully sampled. The interviews were audio recorded, transcribed and analysed thematically.

**Setting:**

Secondary care respiratory unit; community provider of respiratory care; and a clinical commissioning group.

**Participants:**

Nineteen interview participants: nine from the community provider; eight from the hospital and two from the clinical commissioning group.

**Results:**

Staff identified lack of integration between the organisations characterised by: poor communication, lack of trust, absence of shared information technology and ineffective integrative initiatives. The commissioning process created barriers to integration including: contractual limitations which prevented pathway development, absence of agreed clinical governance arrangements and lack of recognition of community work undertaken by hospital staff. Positive working relationships were established over time as staff recognised the skills that each had to offer.

**Conclusions:**

The commissioning process underpinned the relationship between the organisations and contributed to distrust and negative perceptions of the ‘other’. Commissioning an integrated service should incorporate dialogue with stakeholders as early as possible and before the contract is finalised to develop a bedrock of trust.

**Strengths and limitations of this study**

- In-depth interviews with staff from a community healthcare provider, a hospital and the commissioning organisation highlighted barriers and facilitators to establishing an integrated care pathway.
- A range of clinical and non-clinical participants were recruited from organisations involved in the formation and delivery of an integrated respiratory care pathway.
- Interview data reflect a particular time period in the development of the integrated respiratory pathway but the relationship between the organisations was dynamic and evolving.
- The study gives an in-depth account of the experiences of developing and delivering one integrated care service, the findings may not be transferable to other settings.
- The study focuses on staff experiences and does not offer data on patient experiences of the integrated care respiratory pathway.

## Introduction

Integrated care pathways (ICPs) have increasingly been used in healthcare to bring together different organisations associated with the care and treatment of a patient population. ICPs link secondary and primary care and/or organisations from sectors such as social care. The rationale is that integrated care can be ‘the best possible care for the patient, delivered by the most suitable health professional, at the optimal time, in the most suitable setting.’^1^ It also offers the potential for efficiency savings, freeing up clinical resources from hospitals and other healthcare sites. This development of ICPs is central to the UK National Health Service (NHS) Long Term Plan.^2 3^

An integrated respiratory service (IRS) was commissioned by a Clinical Commissioning Group (CCG) in 2016 in a region of the UK to support patients with chronic obstructive pulmonary disease (COPD). We have not named the region to preserve participant anonymity. The service brought together the respiratory department of a secondary care hospital and a not for profit community provider. The rationale of the development was that care could be shared between secondary care and the community to the benefit of patients, who could receive care and support in the community, at the same time reducing demand on secondary care. The development of ICPs for respiratory care is supported by The British Thoracic Society.^1^

The new service commissioned a community provider to deliver four respiratory services: oxygen assessment; pulmonary rehabilitation; hospital admission avoidance and early supported discharge from hospital. The service was integrated in the sense that patients moved between the two organisations at different points of the care pathways. The main points of crossover were joint triaging of referrals for oxygen assessment and pulmonary rehabilitation to assess which patients were appropriate for community care; and phone consultations between the community and the hospital outpatient treatment (HOT) clinic. The HOT clinic was a long-established admission avoidance service used by primary care. It was staffed by a specialist respiratory nurse and consultant respiratory physician. In relation to the new service, it was the main point of contact for community staff who needed clinical advice and support.

A board was established to guide the development of the service at a strategic level and was made up of the commissioning team along with senior clinical and managerial leads from both organisations. There were also joint meetings between clinical staff on a quarterly basis to: forge interworking relationships between staff; and disseminate knowledge and training.

Previous research in relation to integrated respiratory care has identified some promising results in terms of integration and admission avoidance^4–6^ but in general the results have been mixed.^7–9^ Despite the policy prioritisation associated with integrated care, research and evaluations have not produced clear evidence of effectiveness.^3 10–14^ Studies have shown how tensions between the professional cultures of different organisations can impede integration.^15 16^ The commissioning process (the contractual and clinical governance arrangements that underpin collaboration) has also been identified as shaping the working relationships and quality of integration between organisations involved in care pathways.^17^ We undertook a qualitative evaluation of the IRS to understand: (1) the extent to which staff within the IRS worked together to provide an ICP and (2) the relationship between commissioning and the development of an integrated service.

### Patient and public involvement

This study looked at the process of integrating care from the perspective of staff working for the care providers and commissioning organisation. Because of the staff focus we decided that patient and public involvement was not appropriate.

## Methods

The study took place between May 2018 and May 2019 and used semi-structured interviews with participants purposively sampled from the three organisations involved: the secondary care hospital, the community service provider and CCG commissioners. Inclusion criteria were staff who were involved in the formation and delivery of the IRS.

In the hospital and community organisation, study information was distributed by senior staff and potential participants were invited to contact the research team. A reminder email was sent after 2 weeks. Sampling sought to capture the range of roles in the IRS including: senior clinical/management staff, team leads, clinical and support staff. The research team contacted those involved with the commissioning process directly by email and sent them research information inviting them to respond.

All participants gave informed consent. Interviews were conducted by JB and TS and were audio recorded. A topic guide was developed by the research team to support the interviews (see online supplemental file 1) based on the study research questions and issues raised by relevant research literature. The guide was used flexibly and adjusted iteratively as interviews progressed. Data were reviewed throughout the data collection period at research team meetings until we were satisfied that we had sampled relevant staff from both organisations and that no new data were emerging.^18^

10.1136/bmjopen-2020-040267.supp1 Supplementary data

Audio recordings of interviews were transcribed by a professional transcribing service who entered into a confidentiality agreement with the research team and were anonymised and imported into NVivo 11. We used a data driven inductive approach to identify patterns and themes across the dataset and by research site. The data were analysed thematically.^19^ Analysis commenced with open coding. JB and TS each coded a sample of early transcripts and jointly developed an initial coding framework. Following a further round of double coding, the framework was agreed and applied to the full dataset by TS. The analysis proceeded by developing broader categories through comparison across the transcripts and identifying higher-level recurring themes. Members of the study team met regularly to discuss analysis.

The research adhered to the Consolidated criteria for Reporting Qualitative research guidelines for qualitative research (see online supplemental file 2).

10.1136/bmjopen-2020-040267.supp2 Supplementary data

## Results

Nineteen interviews were conducted between October and December 2018, approximately 2 years after the IRS started. Seventeen were face to face and two were over the telephone. Interviews lasted between 14 and 86 min. Details of participants by role are detailed in table 1.

**Table 1:** Participant details

Sector/professional background	Participants
Hospital	
Respiratory nurse	3
Respiratory physiotherapist	2
Respiratory physiotherapist support	1
Respiratory team leads	2
Community provider	
Respiratory nurse	2
Respiratory physiotherapists	2
Nursing support staff	3
Service lead/management	2
Clinical Commissioning Group	
Commissioners	2
Total participants	19
Notes on sectors	
Hospital	NHS Hospital Trust Respiratory department (teaching hospital). Included nursing staff, physiotherapists, team leads and consultants.
Community provider	Participants providing/leading nursing services delivered in patients’ homes, primary care and community settings. Included nursing staff, nursing support staff, physiotherapists and team leads.
Clinical Commissioning Group	Participants were key drivers in the development of the Integrated Respiratory Service and were pivotal figures in establishing the relationships between secondary care and the community provider.

NHS, National Health Service.

We present our findings under two broad themes: whether integration is working; and the impact of commissioning on integration. Quotes are tagged by study ID and organisation. Participant roles are not included to preserve anonymity.

### Integration: is it working?

In this section, we look at the working relationship between staff from both organisations to assess the extent to which they were able to work together effectively to deliver an integrated service to patients.

#### It’s a good idea but …

Participants from both organisations thought the IRS was a good idea and felt the service offered advantages for patients. The pulmonary rehabilitation service offered a choice of location for the 6-week course at either the established hospital service or a new programme located at a sports centre. Staff from both organisations supported patient access to a specialist community team to avoid hospital admission and the assessment of patient oxygen in community settings,

I think the concept of an integrated respiratory team working across primary and secondary care is a great idea but I think there has to be that shift into community care and keeping these patients out of hospital and we have to change the way that we’re thinking about managing these patients. (02-hospital)

I think it’s a good thing. I just think we all need to be consistent in what we do and how we provide it really. (11-community)

Despite this positive perspective, many staff felt that integration had not developed as anticipated, especially joint working relationships between staff from both organisations,

I don’t think we are integrated at all. (14-community)

There has to be a responsibility for the patient but because we’re integrated, they should be able to pass between the two providers seamlessly and sometimes that doesn’t happen. (04-hospital)

#### Barriers to integration: communication, trust and perception of the ‘other’

The lack of integration was evident in the way that the ‘other’ organisation was viewed. There was a perception of imbalance and a lack of reciprocity. The communication between the organisations became blocked and misaligned as these perceptions affected their interorganisational relationships. The hospital staff generally felt that their experience and expertise was not being utilised by the community team,

We’ve always said right from the start, ‘If you’ve ever got any questions or concerns phone us and we’ll do our best to help you.’ That very rarely ever happens, even now it hardly happens. (07-hospital)

Despite this, community staff did not feel encouraged to ask for help,

You can ring a hot consultant but they’re always madly busy because they’ve got a hot clinic. Along with that is the irritation. We’re an irritation. (13-Community)

The perception of imbalance came through in comments from both organisations whereby ‘they’ had obligations or were undertaking actions that the ‘other’ did not reciprocate,

We refer to them, but in the duration of the prevention of hospital admission services running—we’ve only had three referrals from them … (08-community)

If we pick the patients up who are under the team in the community, when they’re in hospital, they would never phone us and say, ‘We’ve sent in Mrs Bloggs today, can you catch up with her?’ There isn’t that sort of communication. They like us to let them know when we’ve caught up with a patient, but they don’t do it reciprocally. (07-hosptital)

There was a sense in the early stages of the service that patients were appropriated as a currency through which either organisation could secure their position or legitimacy. The community organisations perceived that hospital staff dominated the mechanisms of joint triage which gave them control over patient care, whereas hospital staff were concerned that the community organisation would take their patients,

(The Hospital) seem to have a lot of control, they get all the referrals, so we don’t get sight of the referrals they get so it’s like a centralised referral system but they manage it so it doesn’t feel very joint and there’s quite a few processes that are like that with (the Hospital). (17-community)

To start with it just felt, and we were informed that they were being told by senior members of the team to take everybody, any referral had to go to (the community team). It just felt that when they were set up all they wanted to do—the (community) team just wanted to take all the patients from us. (07-hospital)

Members of the community team described the relationship between the organisations in hierarchical terms. At times, they felt they were only able to undertake key aspects of their work with the approval of the hospital team. On the other, hand the hospital team described a feeling of uncertainty about the clinical skills held by the community team and the degree to which they could provide clinically appropriate patient care,

We maintain a professional relationship. We have good conversations with them. A couple of them are a bit patronising. I just let it go over my head and I counsel those that don’t find that very easy, but I suppose my gut feeling is that we all feel a bit substandard and that’s not an integrated team. (13-community)

The general feeling is that the skills in the community team are not as high as the skills and experience within the team sitting in the hospital. (02-hospital)

The perceived skill differential also manifested itself in a lack of trust and confidence in each other and each other’s organisation.

I think one of the biggest barriers to start with was change—change within our team and a change of mindset and actually these patients can be managed in the community (02-hospital)

The final pathway we managed to get up and running was the prevention of admission and we kept going round and round in circles with it and primarily because I think that the team in the hospital didn’t think we had the expertise. (18-community)

#### Information technology systems

Lack of access to a shared patient record system was consistently cited by staff in both organisations as a barrier to integration. A number of workarounds were developed which included community staff being able to log on to the secondary care system but this entailed manually transposing content onto their system which proved to be cumbersome and duplicated community work. The lack of a shared system served to maintain distance between the organisations,

Here, we have access to a computer system and we put a lot of information on there about our patients when we’ve seen them. For early supported discharge, for the respiratory nurse clinics, and to do with oxygen. Whilst the (community) team didn’t have access—not all of them had access to that system … So, straight away, we couldn’t see what they were doing, and they couldn’t see what we were doing. (01-hospital)

The main barrier is the fact we use different note systems. It’s a huge barrier.—either they’ve got read only access but not on all their computers in the hospital so it’s not accessible for them. We use the same systems as the GPs, district nurses, community matrons which has a plethora of information that if when a patient is admitted into hospital they get opened up, it would give them a lot more awareness of what we’ve been trying to do with that patient. (11-community)

#### Integrative mechanisms

There were differing perspectives on the joint staff meetings. The hospital team were more positive whereas community members did not feel that these meetings engaged with the barriers to integrated working,

We have these meetings every three or four months with this team and every time before we go I wish we could have an open and honest conversation about how people feel, but nobody’s ever brave enough to do it, so I’d rather all cards on the table … there have been a few difficult conversations, few snipey remarks. (13-community)

We’ve had good meetings where we’ve talked about maybe issues that we’ve both experienced and we’re maybe prescribing oxygen or you know, certain patients—what do we do about this particular patient you know and that’s been really useful for both sides I think but yes it’s good to have those regular meet ups I think and just discuss the things we come across on a daily basis. (03-hospital)

#### Integrated working: what worked?

Where working relationships did improve this often came about informally. Some nurses were proactive about phoning and emailing colleagues from their partner organisation in cases of uncertainty. More importantly, as staff worked together on joint triage and phone consultations their knowledge of each other and the complimentary skills and experience they brought to the pathway grew, along with developing mutual respect. It was these contacts rather than the more formalised group meetings that enabled a more effective and comfortable level of communication,

Over time obviously the service in secondary care has become more trusting of what we can deliver in the community and trust the fact that if we’ve got any concerns, then we will report those in to the consultant and if necessary, we’ll either arrange for somebody to go to a hot clinic appointment because we can access that by contacting secondary care direct or we will admit. (18-community)

(With) time comes experience and now we’re starting to feel more comfortable with it and we feel that when we have a conversation with, well personally if I’m having a conversation with (the community team) I feel more—you know the conversations are obviously coming from people who’ve got experience now and I think that improves with time really. (04-hospital)

### The commissioning process and integrated working

In this section, we look at the role of the commissioning process to provide context to issues highlighted above.

#### A ‘new’ community service?

The services that the community organisation were commissioned to provide were not completely new. The hospital had been providing similar services, some of them in community settings, prior to the IRS. Pulmonary rehab was run at a community hospital site. There was also an established ‘early supported discharge’ programme involving hospital-based specialist nursing and physiotherapists providing clinical support for patients at home. The commissioning of services already being undertaken by the hospital therefore created confusion, resentment and uncertainty, for both healthcare providers.

Once we started to recruit into the service we then started to find that (the hospital) were saying well why are you recruiting to that when we’ve got that already? So that’s kind of where the…certain amount of confusion started. (18-community)

We were already out in the community and providing oxygen, providing pulmonary rehabilitation and we were providing early supported discharge and then all of a sudden we had a team that were then commissioned to provide the same services. (02-hospital)

This established a foundation of mistrust between the two organisations—the hospital saw the community work they had established being removed from them whereas the community organisation felt the hospital were holding on to sections of the service the community had been commissioned to undertake. Pathways developed and integrated structures were created but the weight of unresolved tensions continued to undermine integration.

I think we all went in with a bit of you know, well we need to protect our end of things. (18-community)

The way it was set up probably wasn’t the most ideal. We originally sat down and we informed commissioners of what we provided but I don’t think that was taken on board at all because when the original business case then came out, saying that this is actually what they wanted (the community team) to provide, was actually everything that we were providing so to start with we’d already kind of got our heckles up a little bit about that process (02-hospital)

#### Commissioning anomalies

The ‘early supported discharge pathway’ element of the new service never started. The contract with the community provider funded care 5 days a week whereas the hospital delivered care 7 days a week which was aligned with the hospital’s early supported discharge pathway. The outcome was that the secondary care hospital remained as provider of early supported discharge work,

It just impacts on the amount of work we can give them as far as an admission avoidance or an early supported discharge pathway was concerned. Because we couldn’t give them a patient on a Thursday because they wouldn’t be able to see them over the weekend, they’d be handing them back for us to look after. (06-hospital)

This limitation served to widen the gulf between the organisations. For the hospital team it fed into a view that the community organisation was not a fully fledged healthcare organisation who could meet the service specifications that they were providing. The community team felt restricted by the contract and unable to prove themselves.

#### Commissioning and clinical governance

Clinical governance,^20^ the framework by which the quality of patient care is assured, was a divisive issue between the organisations. The hospital had an NHS clinical resource in the form of established respiratory consultants, nurses and physiotherapists which formed the bedrock of their clinical supervision. The community organisation had to build their team around their nursing and physiotherapist staff. They did not have the same access to clinical supervision, particularly consultant support. The solution was for community staff to phone HOT clinic clinicians for advice and support when they required it. There was no dedicated time provided for this and the HOT clinic clinicians felt it placed more demand on their time and added a level of uncertainty to their clinical responsibilities,

We get a lot of telephone calls when we are doing our HOT weeks, being asked questions that are really basic… It’s one of the weaknesses I think in this process is (the community team) does not have in place clinical governance arrangements that we would find acceptable (19-hospital)

When we were discussing the service spec with the CCG and the CSU (commissioning support unit) for the service here, we raised the fact that it would need to have an element of clinical supervision from the consultants built into the spec … They didn’t want to allocate any money to that (18-community)

## Discussion

### Summary

The IRS was developed to incorporate aspects of specialist respiratory care into community settings. To work effectively, the new service required close working between staff from the hospital and community providers. This involved joint triage sessions and clinical dialogue between staff. Our study looked at integration from the perspective of staff with a focus on joint working practices. Our results indicate that effective integration between the organisations had not been achieved. There were substantive problems generated by information technology (IT) incompatibility but more significant was the level of mistrust that staff felt towards their partner organisation which undermined effective joint working. Negative perceptions of power, hierarchy and control were expressed by staff from both organisations. However, it was notable how the relationships between organisations in the IRS improved over time. The barriers of mistrust gave way to a growing recognition of the knowledge and skills held by all staff. Interaction became easier, leading to something closer to integrated working.

### Comparison with other literature

There are few qualitative studies of integrated care in relation to respiratory disease^21 22^ and we are not aware of studies that focus on the processes of integration and commissioning. As such, we have situated our findings in the wider literature around integrated care and commissioning. Our findings resonate with other studies,^23 24^ which show support for the principle of an integrated service among staff but with limited evidence that it had been achieved. Staff in the IRS felt there was a hierarchical relationship between organisations which previous studies have identified as disruptive to integrated working. Similarly, interorganisational mistrust was identified as the key barrier to a widespread programme of integration in New Zealand.^25^

We explored the role of commissioning in shaping the relationship between the IRS organisations. The framework of integration is aligned with what a Kings Fund report^26^ identifies as an ‘alliance’ model, whereby a number of providers hold contracts with the commissioning body. They argue that the alliance model requires a high level of trust between organisations to work effectively. Also, creating mechanisms and structures that bring organisations together does not in itself create effective interprofessional relationships.^27^ The joint meetings intended to support integration in the IRS were viewed with a mixture of ambivalence and hostility. Similarly, the contract between organisations should not be seen as the mechanism of integration, ‘the contract is merely the ‘scaffolding’ for the integrated model.’^26^ The Kings Fund argue that the structure of collaborations between healthcare providers needs to be developed between all parties before the contract is finalised.^26^ A similar argument underpinned progress made in New Zealand where they moved away from a top-down approach to one which encouraged collaborations at a grass roots level prior to formal integration as a building block to integrated care.^28^ This resonates with recommendations made following the introduction of integrated care systems in London which argues that commissioners need to engage all stakeholders.^15 29^ They also highlight the need to attend to history which is an issue that slowed the progress of the service in this study.

### Strengths and limitations

In-depth interviews with a range of staff from organisations involved in the IRS enabled understanding of factors that inhibited and facilitated an integrated service. Our findings can support the development of integrated services in other areas but we acknowledge that the data may not be transferable to all settings. The data are taken from a particular time point in the development of the service. Had interviews taken place earlier or later in the process the results may have carried a different emphasis. The study does not evaluate patient outcome and experience. As part of a wider study, a quantitative paper looking at the impact of the IRS on COPD hospital admissions has also been published.^30^ However, further research is needed to capture patient experience of this pathway.

### Implications for policy

Our results highlight challenges and offer lessons at a time when integrated services are being developed throughout the UK. There was a bedrock of good will around the idea of integration among healthcare staff in our study. Commissioners of integrated care can build on such positive orientation through use of precontract partnership building so that providers have opportunities to shape and effectively cocommission a new service. It will also create opportunity to: address structural issues like IT; agree clinical governance arrangements; and engender interorganisational trust.

## Supplementary Material

Reviewer comments

Author's manuscript
