# Supplementary material for: Integrating care between an NHS hospital, a community provider and the role of commissioning: the experience of developing an integrated respiratory service
Source: BMJ Open. 2020 Dec 21;10(12):e040267. doi: 10.1136/bmjopen-2020-040267 (PMC7754656; doi:10.1136/bmjopen-2020-040267)
Supplement: Supplementary data [file bmjopen-2020-040267supp001.pdf]

Supplementary file 1 - Evaluation of the Integrated Respiratory Services (IRS) Interview Topic guide V2 22/06/2018

Part A: Introduction, consent and background

- Thanks, introduce self, re-state purpose of the interview (to understand the development, management and ongoing use of the integrated respiratory pathway (IRS))
- Discussion of how interview will be recorded, right to withdrawal, issues of confidentiality, anonymisation and informed consent (*face-to-face written consent, telephone verbal consent).* Verbal consent: *switch audio recorder on* - For the audio recording, can I check that:
  - You have read and understood the study information sheet?
  - You know that taking part in the interview is voluntary and you are free to stop the interview at any point and you may skip questions you would prefer not to answer?
  - You agree to our conversation being audio recorded?
  - You understand that quotes from the interview may be used to illustrate our findings but it will not be possible to trace who said them?

Part B: Working within the integrated research pathway

- Can you tell us about your role in relation to the integrated respiratory pathway (IRS) and which of the collaborating organisations you work with?
  - Tell us about your experiences of working as part of the IRS
  - How does it differ from the previous pathway of care for respiratory patients
  - What benefits does it offer patients
  - What benefits does it offer for the staff working within the pathway
  - Are there any challenges in working within the pathway
  - Do you perceive any downsides or disadvantages of the pathway for patients
  - Do you perceive any downsides or disadvantages of the pathway for yourself and/or for other members of staff
- The pathway is a collaboration between two different organisations -
  - Tell us about your experience of collaborating and working with the either of these organisations
  - Describe how the relationship has evolved over time
  - What has been the challenges of working with community provider/hospital?
  - What has been the benefits of working with community provider/hospital?
  - Have you trained members of staff at community provider/hospital?
  - Have you received training from members of staff at community provider/hospital?

Part C: Setting up and managing the pathway

- Can you tell us about your role in setting up the pathway
  - Were you involved in the design or establishment of the pathway?
  - What prompted the establishment of the pathway?
  - Can you describe the management and governance structures of the pathway and your role in these?
  - What have been the challenges in setting up and managing the pathway?
  - How have these challenges been overcome?
  - What have been the benefits of managing the pathway (as described)?

Part D: Summary questions

- What do you see as the future for the IRS, how can it be improved are there areas that are problematic?
- Any questions or issues about the IRS that we have not covered
- Thank interviewee for their time and make sure they have our contact details

IRS topic guide 22/06/2018 version 2, IRAS ref: 250022
