# Supplementary material for: Integrating care between an NHS hospital, a community provider and the role of commissioning: the experience of developing an integrated respiratory service
Source: BMJ Open. 2020 Dec 21;10(12):e040267. doi: 10.1136/bmjopen-2020-040267 (PMC7754656; doi:10.1136/bmjopen-2020-040267)
Supplement: Supplementary data [file bmjopen-2020-040267supp002.pdf]

**Supplementary file 2 - COREQ Statement**

# Integrating care between an NHS hospital, a community provider and the role of commissioning: the experience of developing an integrated respiratory service

**Domain 1: Research team and reflexivity**
*Personal Characteristics*
1. Interviewer/facilitator. Which author/s conducted the interview or focus group?
*Jon Banks (JB)*
*Tracey Stone (TS)*

2. Credentials. What were the researcher's credentials? E.g. PhD, MD.
JB*:* PhD
TS: PhD

3. Occupation. What was their occupation at the time of the study?
*JB: Research Fellow*
*TS: Research Associate*

4. Gender. Was the researcher male or female?
*TS - Female.*
*JB - Male*

5. Experience and training. What experience or training did the researcher have?
JB: *Post graduate diploma in social science research methods as part of PhD, experience of collecting and analysing qualitative data with 17 years as a research associate and research fellow.*

*TS: PhD in Research Ethics, 5 years (local and regional) Research Ethics Committee service, Extensive training in qualitative research methods (PhD and subsequent courses) and 15 years' experience conducting qualitative research.*

*Relationship with participants*
6. Relationship established. Was a relationship established prior to study commencement?
*No direct relationship was established with the research participants prior to recruitment.*

7. Participant knowledge of the interviewer. What did the participants know about the researcher? e.g. personal goals, reasons for doing the research?
*The personal goals of the researchers were to complete the aims and objectives of the study only. They were introduced to potential participants by email from the Consultant service lead in the hospital, also co-PI on the project, including full Participant Information which emphasised that taking part in an interview was voluntary. Likewise, in the community setting, the service lead agreed to distribute information to staff. The researchers were not known to the participants prior to the study interviews. When undertaking interviews*

*researchers gave full details about the research along with printed study materials before gaining informed consent. The researchers made their academic standpoint clear to interviewees so they were aware that researchers were from an independent research body not linked to their employers.*

8. Interviewer characteristics. What characteristics were reported about the interviewer/facilitator? e.g. Bias, assumptions, reasons and interests in the research topic.
*The interviewers are social scientists with many years' experience conducting qualitative research on a range of health topics. This research topic was proposed by Academic Clinical collaborators, and not selected by the interviewers due to any particular prior interests or goals related to this research topic.*

**Domain 2: study design**
*Theoretical framework*
9. Methodological orientation and Theory. What methodological orientation was stated to underpin the study? e.g. grounded theory, discourse analysis, ethnography, phenomenology, content analysis
*In the Methods section we explain how we used semi-structured interviews using thematic analysis with NVivo11 software, this was informed by grounded theory and the constant comparison method.*

*Participant selection*
10. Sampling. How were participants selected? e.g. purposive, convenience, consecutive, snowball.
*Purposive sampling, when clinically convenient, was used to select interviewees in order to attempt to capture maximum variation in views and experiences in order that they adequately reflect those of a range of staff involved in the development, implementation and on-going work in the Integrated Respiratory Service.*

11. Method of approach. How were participants approached? e.g. face-to-face, telephone, mail, email
*Please see item 7 above; moreover, potential participants responded to the initial information from the service leads in both organisations by contacting the researchers directly to discuss the project and/or arrange an interview date and time. This took place at or near the participants' places of work for their convenience. All interviewees gave written informed consent.*

12. Sample size. How many participants were in the study?
*We interviewed 19 members of staff, 8 from the secondary care hospital, 9 from the community provider and 2 who were members of the clinical commissioning group.*

13. Non-participation. How many people refused to participate or dropped out? Reasons
*Only one potential participant was eventually not interviewed. Whilst she was willing, after 3 attempts to meet, clinical duties and shift patterns eventually prevented this. This took place towards the end of the study when data saturation was occurring but other interviews*

*were still taking place. For this reason, as well as to avoid inappropriate pressure on the participant the interview did not take place.*

14. Setting of data collection. Where was the data collected? e.g. home, clinic, workplace
*Data collection took place at or near the participants' place of work, at their preference and convenience.*

15. Presence of non-participants. Was anyone else present besides the participants and researchers?
*These were one to one face to face interviews held in private or in quiet public places, (local café in the community and staff social area which was large enough to ensure privacy and where the participants felt comfortable to express their opinions).*

16. Description of sample. What are the important characteristics of the sample? e.g. demographic data, date.
*All the staff interviewed were involved in the development, implementation and on-going operation of the Integrated Respiratory Service. Interview participants were purposively sampled and recruited to cover the range of staff who worked with the service. (See table 1 in paper)*

*Data collection*
17. Interview guide. Were questions, prompts, guides provided by the authors? Was it pilot tested?
*A topic guide was used for all interviews (provided as an additional file). The focus of the topic guide was participants' views and experiences of the Integrated Respiratory Service and was modified as data collection progressed. The topic guide was used flexibly so that interviewees could raise other issues they felt were important whilst also allowing in-depth exploration of emergent themes.*

18. Repeat interviews.
*We did not conduct repeat interviews*

19. Audio/visual recording. Did the research use audio or visual recording to collect the data?
*Yes, all interviews were audio recorded, with the informed consent of participants.*

20. Field notes. Were field notes made during and/or after the interview or focus group?
*Field notes were not made during this study. Reflexive notes were made on password protected computers to aid the iterative development of the topic guide.*

21. Duration. What was the duration of the interviews or focus group?
*Between 14 and 86 minutes*

22. Data saturation. Was data saturation discussed?
*Yes, this was discussed in team meetings.*

23. Transcripts returned. Were transcripts returned to participants for comment and/or correction?
*No.*

**Domain 3: analysis and findings**
*Data analysis*
24. Number of data coders. How many data coders coded the data?
*Two. Data was coded by the researcher Tracey Stone, and Jon Banks, a senior qualitative research fellow. The coding frame was developed by JB and TS following the initial double coding of four interviews covering a range of staff. Coding and iterative developments were discussed at regular meetings.*

25. Description of the coding tree. Did authors provide a description of the coding tree?
*No.*

26. Derivation of themes. Were themes identified in advance or derived from the data?
*Themes were derived deductively from our research questions and inductively from the data.*

27. Software. What software, if applicable, was used to manage the data?
*We used NVivo 11 qualitative software package to manage the data.*

28. Participant checking. Did participants provide feedback on the findings?
*No.*

29. Quotations presented. Were participant quotations presented to illustrate the themes / findings? Was each quotation identified? e.g. participant number
*Yes, with quotations identified using alphanumeric labels for the site, staff role and interview number. These categories are intentionally quite broad. Whilst readers may appreciate more specific information for context, the sample size and variation of roles within the sample in both organisations, would undermine anonymity.*

30. Data and findings consistent. Was there consistency between the data presented and the findings?
*Yes.*

31. Clarity of major themes. Were major themes clearly presented in the findings?
*Yes.*

32. Clarity of minor themes. Is there a description of diverse cases or discussion of minor themes?
*Yes.*
