# Supplementary material for: Integrating care between an NHS hospital, a community provider and the role of commissioning: the experience of developing an integrated respiratory service
Source: BMJ Open. 2020 Dec 21;10(12):e040267. doi: 10.1136/bmjopen-2020-040267 (PMC7754656; doi:10.1136/bmjopen-2020-040267)
Supplement: Author's manuscript [file bmjopen-2020-040267.draft_revisions.pdf]

BMJ Open

BMJ Open is committed to open peer review. As part of this commitment we make the peer review history of every article we publish publicly available.

When an article is published we post the peer reviewers' comments and the authors' responses online. We also post the versions of the paper that were used during peer review. These are the versions that the peer review comments apply to.

The versions of the paper that follow are the versions that were submitted during the peer review process. They are not the versions of record or the final published versions. They should not be cited or distributed as the published version of this manuscript.

BMJ Open is an open access journal and the full, final, typeset and author-corrected version of record of the manuscript is available on our site with no access controls, subscription charges or pay-per-view fees (http://bmjopen.bmj.com).

If you have any questions on BMJ Open's open peer review process please email info.bmjopen@bmj.com

BMJ Open

# Integrating care between an NHS hospital, a community provider and the role of commissioning: the experience of developing an integrated respiratory service

| | |
|---|---|
| Journal: | *BMJ Open* |
| Manuscript ID | bmjopen-2020-040267 |
| Article Type: | Original research |
| Date Submitted by the Author: | 12-May-2020 |
| Complete List of Authors: | Banks, Jonathan; National Institute for Health Research Applied Research Collaboration West, University Hospitals Bristol NHS Foundation Trust ; University of Bristol, School of Population Health Sciences, Bristol Medical School<br>Stone, Tracey; National Institute for Health Research Applied Research Collaboration West, University Hospitals Bristol ; University of Bristol, School of Population Health Sciences, Bristol Medical School<br>Dodd, James; University of Bristol, Academic respiratory unit, Southmead Hospital |
| Keywords: | Organisation of health services < HEALTH SERVICES ADMINISTRATION & MANAGEMENT, QUALITATIVE RESEARCH, Adult thoracic medicine < THORACIC MEDICINE, Health policy < HEALTH SERVICES ADMINISTRATION & MANAGEMENT |
| | |

SCHOLARONE™
Manuscripts

*I, the Submitting Author has the right to grant and does grant on behalf of all authors of the Work (as defined in the below author licence), an exclusive licence and/or a non-exclusive licence for contributions from authors who are: i) UK Crown employees; ii) where BMJ has agreed a CC-BY licence shall apply, and/or iii) in accordance with the terms applicable for US Federal Government officers or employees acting as part of their official duties; on a worldwide, perpetual, irrevocable, royalty-free basis to BMJ Publishing Group Ltd ("BMJ") its licensees and where the relevant Journal is co-owned by BMJ to the co-owners of the Journal, to publish the Work in this journal and any other BMJ products and to exploit all rights, as set out in our licence.*

*The Submitting Author accepts and understands that any supply made under these terms is made by BMJ to the Submitting Author unless you are acting as an employee on behalf of your employer or a postgraduate student of an affiliated institution which is paying any applicable article publishing charge ("APC") for Open Access articles. Where the Submitting Author wishes to make the Work available on an Open Access basis (and intends to pay the relevant APC), the terms of reuse of such Open Access shall be governed by a Creative Commons licence – details of these licences and which Creative Commons licence will apply to this Work are set out in our licence referred to above.*

*Other than as permitted in any relevant BMJ Author's Self Archiving Policies, I confirm this Work has not been accepted for publication elsewhere, is not being considered for publication elsewhere and does not duplicate material already published. I confirm all authors consent to publication of this Work and authorise the granting of this licence.*

For peer review only

# Integrating care between an NHS hospital, a community provider and the role of commissioning: the experience of developing an integrated respiratory service

**Authors**
Jon Banks[1,2] https://orcid.org/0000-0002-3889-6098
Tracey Stone[1,2] https://orcid.org/0000-0003-2627-3843
James W Dodd[3] https://orcid.org/0000-0003-4805-5759

**Affiliations**
1. The National Institute for Health Research, Applied Research Collaboration West (NIHR ARC West), University Hospitals Bristol NHS Foundation Trust, BS1 2NT Bristol UK
2. Population Health Sciences, Bristol Medical School, University of Bristol, UK BS8 1UD
3. Academic respiratory unit, University of Bristol, Southmead Hospital Bristol, UK BS10 5NB

**Corresponding author**
Jon Banks
9th Floor, Whitefriars, Lewins Mead,
Bristol, United Kingdom BS1 2NT
Email: Jon.Banks@Bristol.ac.uk
Tel: 0117 342 1244

**Word count**
3999

**Key Words**
Delivery of health care, integrated; qualitative research; pulmonary disease, chronic obstructive

## Abstract

### Objectives

A regional integrated respiratory service was commissioned in 2016 in the UK to support patients with chronic obstructive pulmonary disease. The service brought together the respiratory department of a National Health Service hospital and a not-for-profit community provider. This paper evaluates: (a) the perceived efficacy of integrated working between the organisations from the perspective of staff; and (b) the relationship between commissioning and integration of the services.



For peer review only - http://bmjopen.bmj.com/site/about/guidelines.xhtml

## Design

Semi-structured interviews with staff from the three organisations involved in the integrated respiratory service. Staff were purposefully sampled. The interviews were audio recorded, transcribed and analysed thematically.

## Setting

Secondary care respiratory unit; community provider of respiratory care; and a clinical commissioning group.

## Participants

Nine members of staff were interviewed from the community provider; eight from the hospital; and two from the clinical commissioning group.

## Results

Data showed support for the idea of integration but staff did not feel the organisations were integrated in their delivery of the service. The lack of integration was evident: in the lack of a shared IT platform; poor communication between staff; a perception of inter-organisational hierarchy; lack of trust and negative perceptions of the 'other' organisation that were held by both providers of care.

## Conclusions

The roots of mistrust and the negative perceptions of the 'other' organisation were located in the commissioning process which generated feelings of insecurity on both sides. Commissioning an integrated service should incorporate dialogue with stakeholders as early as possible and before the contract is finalised to develop a bedrock of trust.


# Strengths and limitations of this study

- In depth interviews with staff from a community health care provider and a hospital highlighted barriers and facilitators to establishing an integrated care pathway.

For peer review only

- A range of clinical and non-clinical participants were recruited from organisations involved in the formation and delivery of an integrated respiratory care pathway.
- Interview data reflect a particular time period in the development of the integrated respiratory pathway but the relationship between the organisations was dynamic and evolving.
- The study gives an in-depth account of the experiences of developing and delivering one integrated care service, the findings may not be transferable to other settings.
- The study focuses on staff experiences and does not offer data on patient experiences of the integrated care respiratory pathway.

Word count – 3999

For peer review only

## Introduction

Integrated care pathways (ICPs) have increasingly been used in healthcare to bring together different organisations associated with the care and treatment of a patient population. ICPs link secondary and primary care and/or organisations from sectors such as social care. The rationale is that integrated care can be '*the best possible care for the patient, delivered by the most suitable health professional, at the optimal time, in the most suitable setting.'* [1] It also offers the potential for efficiency savings, freeing up clinical resources from hospitals and other health care sites.

This development of ICPs as a priority of the UK and international policy agenda is central to the UK National Health Service Long Term Plan.[2, 3] Despite this policy prioritisation, research and evaluations have not produced clear evidence of effectiveness.[4-6]

An integrated respiratory service (IRS) was commissioned by a clinical commissioning group (CCG) in 2016 in a region of the UK to support patients with chronic obstructive pulmonary disease (COPD). The service brought together the respiratory department of a secondary care hospital and a not for profit community provider. The rationale was that, where



For peer review only - http://bmjopen.bmj.com/site/about/guidelines.xhtml

clinically appropriate, more care could be undertaken in the community to the benefit of patients and at the same time reduce demand on secondary care.

The new service commissioned a community provider to deliver four respiratory services: oxygen assessment; pulmonary rehabilitation; hospital admission avoidance; and early supported discharge from hospital. The service was integrated in the sense that patients moved between the two organisations at different points of the care pathways. The main points of crossover were joint triaging of referrals for oxygen assessment and pulmonary rehabilitation to assess which patients were appropriate for community care; and phone consultations between the community and the Hospital Outpatient Treatment (HOT) clinic. The HOT clinic was a long-established admission avoidance service used by primary care. It was staffed by a specialist respiratory nurse and consultant respiratory physician. In relation to the new service it was the main point of contact for community staff who needed clinical advice and support.

We undertook an evaluation of the IRS using quantitative and qualitative methods. Previous research in this area has found some promising results in terms of integration and admission avoidance[7, 8] but in general the results for integrated respiratory care are mixed.[9-12] We report the qualitative study here to examine: (a) the perceived efficacy of integrated working between the organisations from the perspective of staff; and (b) the relationship between commissioning and integration of the services. We focus on the former because studies have highlighted the role of professional and organisational culture[13, 14] in shaping integrated pathways, the latter because the commissioning role has been identified as the driving force in the formation of integrated care.[15]



For peer review only - http://bmjopen.bmj.com/site/about/guidelines.xhtml

## Patient involvement

This study looked at the process of integrating care from the perspective of staff working for the care providers and commissioning organisation. Because of the staff focus we decided that patient involvement was not appropriate.

## Methods

This study used semi-structured interviews with participants purposively sampled from the three organisations involved: the secondary care hospital, the community service provider and CCG commissioners.

In the hospital and community organisation, study information was distributed by senior staff and potential participants were invited to contact the research team. A reminder email was sent after 2 weeks. Sampling sought to capture the range of roles in the IRS including: senior clinical/management staff, team leads, clinical staff and support staff. The research team contacted those involved with the commissioning process directly by email and sent them research information inviting them to respond.

All participants gave informed consent and interviews were audio-recorded. A topic guide was developed by the research team to support the interviews (supplementary file 1) based on the study research questions and issues raised by relevant research literature. The guide was used flexibly and adjusted iteratively as interviews progressed. Data were reviewed throughout the data collection period at research team meetings until we were satisfied that we had sampled relevant staff from both organisations and that no new data were emerging.[16]

Audio recordings of interviews were transcribed, anonymised and imported into NVivo11. We used a data driven inductive approach to identify patterns and themes across the



For peer review only - http://bmjopen.bmj.com/site/about/guidelines.xhtml

dataset and by research site. The data were analysed thematically.[17] Analysis commenced with open coding. JB and TS each coded a sample of early transcripts and jointly developed an initial coding framework. Following a further round of double coding, the framework was agreed and applied to the full dataset by TS. The analysis proceeded by developing broader categories through comparison across the transcripts and identifying higher-level recurring themes. Members of the study team met regularly to discuss analysis.

The research was approved by a UK University Research Ethics Committee and the Health Research Authority in England. It adhered to the COREQ guidelines for qualitative research (supplementary file 2).

## Results

Nineteen interviews were conducted between October and December 2018. Seventeen were face-to-face and two were over the telephone. Interviews lasted between 14-86 minutes. Details of participants by role are detailed in table 1.

## Table 1: participant details

| **Sector / professional background** | **Participants** |
|---|---|
| ***Hospital*** | |
| Respiratory nurse | 3 |
| Respiratory physiotherapist | 2 |
| Respiratory physiotherapist support | 1 |
| Respiratory team leads | 2 |
| ***Community Provider*** | |
| Respiratory nurse | 2 |
| Respiratory physiotherapists | 2 |
| Allied organisational staff | 3 |
| Service lead/management | 2 |
| ***Clinical Commissioning Group*** | |
| Commissioners | 2 |
| **Total participants** | **19** |



For peer review only - http://bmjopen.bmj.com/site/about/guidelines.xhtml

| Notes on sectors | |
|---|---|
| *Hospital* | NHS Hospital Trust Respiratory department (teaching hospital). Included nursing staff, physiotherapists, team leads and consultants. |
| *Community provider* | Participants providing/leading nursing services delivered in patients' homes, primary care and community settings. Included nursing staff, nursing support staff, physiotherapists and team leads. |
| *Clinical Commissioning Group* | Participants were key drivers in the development of the Integrated Respiratory Service (IRS) and were pivotal figures in establishing the relationships between secondary care and the community provider. |

For peer review only

We present our findings under two broad themes: whether integration is working; and the impact of commissioning on integration. Quotes are tagged by study ID and organisation. Participant roles are not included to preserve anonymity.

## Integration – is it working?

In this section we look at the working relationship between staff from both organisations to assess the extent to which they were able to work together effectively to deliver an integrated service to patients.

### It's a good idea but …

Participants from both organisations thought the IRS was a good idea and felt the service offered advantages for patients. The pulmonary rehabilitation service offered a choice of location for the six-week course at either the established hospital service or a new programme located at a sports centre. Staff from both organisations supported patient access to a specialist community team to avoid hospital admission and the assessment of patient oxygen in community settings,

> *I think the concept of an integrated respiratory team working across primary and secondary care is a great idea but I think there has to be that shift into community care and keeping these patients out of hospital and we have to change the way that we're thinking about managing these patients.*
> ***(02- hospital)***



For peer review only - http://bmjopen.bmj.com/site/about/guidelines.xhtml

*I think it's a good thing. I just think we all need to be consistent in what we do and how we provide it really.* ***(11-community)***

Despite this positive perspective, many staff felt that integration had not developed as anticipated, especially joint working relationships between staff from both organisations,

*I don't think we are integrated at all.* ***(14-community)***

*There has to be a responsibility for the patient but because we're integrated, they should be able to pass between the two providers seamlessly and sometimes that doesn't happen.* ***(04-hospital)***

## Barriers to integration – communication, trust and perception of the 'other'

The lack of integration was evident in the way that the 'other' organisation was viewed. There was a perception of imbalance and a lack of reciprocity. The communication between the organisations became blocked and misaligned as these perceptions affected their inter-organisational relationships. The hospital staff generally felt that their experience and expertise was not being utilised by the community team,

*We've always said right from the start, "If you've ever got any questions or concerns phone us and we'll do our best to help you." That very rarely ever happens, even now it hardly happens.* ***(07-hospital)***

Despite this, community staff did not feel encouraged to ask for help,

*You can ring a hot consultant but they're always madly busy because they've got a hot clinic. Along with that is the irritation. We're an irritation.* ***(13-Community)***

The perception of imbalance came through in comments from both organisations whereby 'they' had obligations or were undertaking actions that the 'other' did not reciprocate,

*We refer to them, but in the duration of the prevention of hospital admission services running – we've only had three referrals from them …* ***(08-community)***

*If we pick the patients up who are under the team in the community, when they're in hospital, they would never phone us and say, "We've sent in Mrs Bloggs today, can you catch up with her?" There isn't that sort of communication. They like us to let them know when we've caught up with a patient, but they don't do it reciprocally.* ***(07-hosptital)***

There was a sense in the early stages of the service that patients were appropriated as a currency through which either organisation could secure their position or legitimacy. The



For peer review only - http://bmjopen.bmj.com/site/about/guidelines.xhtml

community organisations perceived that hospital staff dominated the mechanisms of joint triage which gave them control over patient care, whereas hospital staff were concerned that the community organisation would take their patients,

> *[The Hospital] seem to have a lot of control, they get all the referrals, so we don't get sight of the referrals they get so it's like a centralised referral system but they manage it so it doesn't feel very joint and there's quite a few processes that are like that with [the Hospital].* ***(17-community)***

> *To start with it just felt, and we were informed that they were being told by senior members of the team to take everybody, any referral had to go to [the community team]. It just felt that when they were set up all they wanted to do – the [community] team just wanted to take all the patients from us.* ***(07-hospital)***

Members of the community team described the relationship between the organisations in hierarchical terms. At times, they felt they were only able to undertake key aspects of their work with the approval of the hospital team. On the other hand the hospital team described a feeling of uncertainty about the clinical skills held by the community team and the degree to which they could provide clinically appropriate patient care,

> *We maintain a professional relationship. We have good conversations with them. A couple of them are a bit patronising. I just let it go over my head and I counsel those that don't find that very easy, but I suppose my gut feeling is that we all feel a bit substandard and that's not an integrated team.* ***(13-community)***

> *The general feeling is that the skills in the community team are not as high as the skills and experience within the team sitting in the hospital.* ***(02-hospital)***

The perceived skill differential also manifested itself in a lack of trust and confidence in each other and each other's organisation,

> *I think one of the biggest barriers to start with was change – change within our team and a change of mindset and actually these patients can be managed in the community* ***(02-hospital)***

> *The final pathway we managed to get up and running was the prevention of admission and we kept going round and round in circles with it and primarily because I think that the team in the hospital didn't think we had the expertise.* ***(18-community)***

For peer review only

## IT systems

Lack of access to a shared patient record system was consistently cited by staff in both organisations as a barrier to integration. A number of workarounds were developed which included community staff being able to log on to the secondary care system but this entailed manually transposing content onto their system which proved to be cumbersome and duplicated community work. The lack of a shared system served to maintain distance between the organisations,

> *Here, we have access to a computer system and we put a lot of information on there about our patients when we've seen them. For early supported discharge, for the respiratory nurse clinics, and to do with oxygen. Whilst the [community] team didn't have access – not all of them had access to that system … So, straight away, we couldn't see what they were doing, and they couldn't see what we were doing.* (***01-hospital)***

> *The main barrier is the fact we use different note systems. It's a huge barrier. – either they've got read only access but not on all their computers in the hospital so it's not accessible for them. We use the same systems as the GPs, district nurses, community matrons which has a plethora of information that if when a patient is admitted into hospital they get opened up, it would give them a lot more awareness of what we've been trying to do with that patient.* ***(11-community)***

## Integrative mechanisms

A board was established to guide the development of the service at a strategic level and was made up of the commissioning team along with senior clinical and managerial leads from both organisations. There were also joint meetings between clinical staff on a quarterly basis, the remit of this group was to forge interworking relationships between staff and to disseminate knowledge and training. The data indicated differing perspectives from staff. The hospital team were more positive whereas community members did not feel that these meetings engaged with the barriers to integrated working,

> *We have these meetings every three or four months with this team and every time before we go I wish we could have an open and honest conversation about how people feel, but nobody's ever brave enough to do it, so I'd rather all cards on the table … there have been a few difficult conversations, few snipey remarks.* ***(13-community)***



For peer review only - http://bmjopen.bmj.com/site/about/guidelines.xhtml

*We've had good meetings where we've talked about maybe issues that we've both experienced and we're maybe prescribing oxygen or you know, certain patients – what do we do about this particular patient you know and that's been really useful for both sides I think but yes it's good to have those regular meet ups I think and just discuss the things we come across on a daily basis.* ***(03-hospital)***

### Integrated working – what worked?

Where working relationships did improve this often came about informally. Some nurses were proactive about phoning and emailing colleagues from their partner organisation in cases of uncertainty. More importantly, as staff worked together on joint triage and phone consultations their knowledge of each other and the complimentary skills and experience they brought to the pathway grew, along with developing mutual respect. Communication became more comfortable and effective,

*Over time obviously the service in secondary care has become more trusting of what we can deliver in the community and trust the fact that if we've got any concerns, then we will report those in to the consultant and if necessary, we'll either arrange for somebody to go to a hot clinic appointment because we can access that by contacting secondary care direct or we will admit.* ***(18-community)***

*[With] time comes experience and now we're starting to feel more comfortable with it and we feel that when we have a conversation with, well personally if I'm having a conversation with [the community team] I feel more – you know the conversations are obviously coming from people who've got experience now and I think that improves with time really.* ***(04-hospital)***

## The commissioning process and integrated working

In this section we look at the role of the commissioning process to provide context to issues highlighted above.

### A 'new' community service?

The services that the community organisation were commissioned to provide were not completely new. The hospital had been providing similar services, some of them in community settings, prior to the IRS. Pulmonary rehab was run at a community hospital site. There was also an established 'early supported discharge' programme involving hospital-based specialist nursing and physiotherapists providing clinical support for patients at home.



For peer review only - http://bmjopen.bmj.com/site/about/guidelines.xhtml

The commissioning of services already being undertaken by the hospital therefore created confusion, resentment and uncertainty, for both healthcare providers,

> *Once we started to recruit into the service we then started to find that [the hospital) were saying well why are you recruiting to that when we've got that already? So that's kind of where the…certain amount of confusion started.* ***(18-community)***
>
> *We were already out in the community and providing oxygen, providing pulmonary rehabilitation and we were providing early supported discharge and then all of a sudden we had a team that were then commissioned to provide the same services.* ***(02-hospital)***

This established a foundation of mistrust between the two organisations – the hospital saw the community work they had established being removed from them whereas the community organisation felt the hospital were holding on to sections of the service the community had been commissioned to undertake. Pathways developed and integrated structures were created but the weight of unresolved tensions continued to undermine integration,

> *I think we all went in with a bit of you know, well we need to protect our end of things.* ***(18-community)***
>
> *The way it was set up probably wasn't the most ideal. We originally sat down and we informed commissioners of what we provided but I don't think that was taken on board at all because when the original business case then came out, saying that this is actually what they wanted [the community team] to provide, was actually everything that we were providing so to start with we'd already kind of got our heckles up a little bit about that process* ***(02-hospital)***

## Commissioning anomalies

The 'early supported discharge pathway' element of the new service never started. The contract with the community provider funded care five days a week whereas the hospital delivered care seven days a week which was aligned with the hospital's early supported discharge pathway. The outcome was that the secondary care hospital remained as provider of early supported discharge work,

> *It just impacts on the amount of work we can give them as far as an admission avoidance or an early supported discharge pathway was concerned. Because we couldn't give them a patient on a Thursday*



For peer review only - http://bmjopen.bmj.com/site/about/guidelines.xhtml

> *because they wouldn't be able to see them over the weekend, they'd be handing them back for us to look after.* ***(06-hospital)***

This limitation served to widen the gulf between the organisations. For the hospital team it fed into a view that the community organisation was not a fully-fledged health care organisation who could meet the service specifications that they were providing. The community team felt restricted by the contract and unable to prove themselves.

### Commissioning and clinical governance

Clinical governance,[18] the framework by which the quality of patient care is assured, was a divisive issue between the organisations. The hospital had an NHS clinical resource in the form of established respiratory consultants, nurses and physiotherapists which formed the bedrock of their clinical supervision. The community organisation had to build their team around their nursing and physiotherapist staff. They did not have the same access to clinical supervision, particularly consultant support. The solution was for community staff to phone HOT clinic clinicians for advice and support when they required it. There was no dedicated time provided for this and the HOT clinic clinicians felt it placed more demand on their time and added a level of uncertainty to their clinical responsibilities,

> *We get a lot of telephone calls when we are doing our HOT weeks, being asked questions that are really basic… It's one of the weaknesses I think in this process is [the community team] does not have in place clinical governance arrangements that we would find acceptable* ***(19-hospital)***

> *When we were discussing the service spec with the CCG and the CSU for the service here, we raised the fact that it would need to have an element of clinical supervision from the consultants built into the spec … They didn't want to allocate any money to that* ***(18-community)***

## Discussion

The IRS was developed to incorporate aspects of specialist respiratory care into community settings. The aim was to offer accessibility to patients, add capacity to respiratory care in



For peer review only - http://bmjopen.bmj.com/site/about/guidelines.xhtml

the community and reduce demand on secondary care. To work effectively, the new service required close working between staff from the hospital and community providers. This involved joint triage sessions and clinical dialogue between staff. Our study looked at integration from the perspective of staff with a focus on joint working practices. Our results resonate with other studies[19, 20] which highlighted support for the principle of an integrated service among staff but little evidence that it had been successfully achieved.

There were substantive problems generated by IT incompatibility but more significant was the level of mistrust that staff felt toward their partner organisation which undermined effective joint working. Negative perceptions of power, hierarchy and control were expressed by staff from both organisations. Community staff felt the hospital team held power through control over the referral system. However, the hospital saw the community organisation as replacing services they were already providing, with additional uncertainty around clinical governance. Studies have highlighted the problem of partnership cultures which lead to hierarchical mechanisms and one partner being privileged over another.[19, 21] Whether such relationships existed in the IRS is not the issue, staff perceived them to be present and disruptive to integrated working. Similarly, inter-organisational mistrust was identified as the key barrier to a widespread programme of integration in New Zealand.[22]

Our data suggest that the commissioning process laid the building blocks of mistrust and undermined integration through: a failure to recognise the established community based work provided by the hospital; the lack of a funded clinical governance resource for the community provider; and contractual issues limiting the community organisation from developing the 'early supported discharge' pathway.

For peer review only



For peer review only - http://bmjopen.bmj.com/site/about/guidelines.xhtml

A Kings Fund report[23] identifies the framework of integration employed for the IRS as an 'alliance' model whereby a number of providers hold contracts with the commissioning body. They argue that the alliance model requires a high level of trust between organisations to work effectively and creating mechanisms and structures that bring organisations together does not in itself create effective interprofessional relationships.[24] The joint meetings intended to support integration in the IRS were viewed with a mixture of ambivalence and hostility. Similarly, the contract between organisations should not be seen as the mechanism of integration, '*the contract is merely the 'scaffolding' for the integrated model*.'[23] The Kings Fund argue that the structure of collaborations between health care providers needs to be developed between all parties before the contract is finalised.[23] A similar argument underpinned progress made in New Zealand where they moved away from a top-down approach to one which encouraged collaborations at a grass roots level prior to formal integration as a building block to integrated care.[25] This resonates with recommendations made following the introduction of integrated care systems in London which argues that commissioners need to engage all stakeholders.[13, 26] They also highlight the need to attend to history which is an issue that slowed the progress of the service in this study.

The data are taken from a particular time point in the development of the service. Had interviews taken place earlier or later in the process the results may have carried a different emphasis. However, it is notable how the relationships between organisations in the IRS improved over time. The barriers of mistrust and the negative perceptions of the other gave way to a growing recognition of the knowledge and skills held by all the staff. Interaction became easier, leading to something much closer to integrated working. This demonstrates



For peer review only - http://bmjopen.bmj.com/site/about/guidelines.xhtml

that if inter-organisational dialogue could have been established earlier it may have reduced or even prevented the mistrust that undermined collaborative working in the IRS.

The study does not evaluate patient outcome and experience. As part of a wider study, a quantitative paper has been submitted for publication elsewhere looking at clinical outcome data. However, further research is needed to capture patient experience of this pathway.

Our results highlight challenges and offer lessons at a time when integrated services are being developed throughout the UK, a move endorsed by The British Thoracic Society.[1] There was a bedrock of good will around the idea of integration among health care staff in our study. Commissioners can build upon such positive orientation through use of pre-contract partnership building so that providers have opportunities to shape and effectively co-commission a new service. It will also create opportunity to: address structural issues like IT; agree clinical governance arrangements; and engender inter-organisational trust.

## References

1. British Thorasic Society. Position statement: integrated respiratory care 2019. 2019. British Thorasic Society. https://www.brit-thoracic.org.uk/document-library/governance-and-policy-documents/position-statements/bts-position-statement-integrated-respiratory-care-2019/ (accessed 08/05/2020)
2. NHS. Long Term Plan 2019. https://www.longtermplan.nhs.uk/wp-content/uploads/2019/08/nhs-long-term-plan-version-1.2.pdf (accessed 08/05/2020)
3. Baxter S, Johnson M, Chambers D, et al. The effects of integrated care: a systematic review of UK and international evidence. *BMC health services research* 2018; 18: 350. 2018/05/12. DOI: 10.1186/s12913-018-3161-3.
4. Georghiou T and Keeble E. Age UK's Personalised Integrated Care Programme. Evaluation of impact on Hospital Activity. Nuffield Trust 31/01/2019. https://www.nuffieldtrust.org.uk/research/age-uk-s-personalised-integrated-care-programme-evaluation-of-impact-on-hospital-activity (accessed 08/05/2020)
5. Georghiou T. Little evidence that integrated care initiatives deliver. *Health Services Journal* 31/01/2019 2019. https://www.hsj.co.uk/integration/little-evidence-that-integrated-care-initiatives-deliver/7024298.article (accessed 08/05/2020)



For peer review only - http://bmjopen.bmj.com/site/about/guidelines.xhtml

6. National Audit Office. *Health and social care integration*. 2017. https://www.nao.org.uk/wp-content/uploads/2017/02/Health-and-social-care-integration.pdf (accessed 08/05/2020)
7. Casas A, Troosters T, Garcia-Aymerich J, et al. Integrated care prevents hospitalisations for exacerbations in COPD patients. *The European respiratory journal* 2006; 28: 123-130. 2006/04/14. DOI: 10.1183/09031936.06.00063205.
8. Gillett K, Lippiett K, Astles C, et al. Managing complex respiratory patients in the community: an evaluation of a pilot integrated respiratory care service *BMJ Open Respiratory Research*. 2016.; 3: e000145. DOI: 10.1136/bmjresp-2016-000145 %J.
9. Boland MR, Tsiachristas A, Kruis AL, et al. The health economic impact of disease management programs for COPD: a systematic literature review and meta-analysis. *BMC pulmonary medicine* 2013; 13: 40. 2013/07/04. DOI: 10.1186/1471-2466-13-40.
10. Kruis AL, Boland MRS, Assendelft WJJ, et al. Effectiveness of integrated disease management for primary care chronic obstructive pulmonary disease patients: results of cluster randomised trial *British Medical Journal*. 2014; 349: g5392. DOI: 10.1136/bmj.g5392 %J BMJ : British Medical Journal.
11. Kruis AL and Chavannes NH. Potential benefits of integrated COPD management in primary care. *Monaldi archives for chest disease = Archivio Monaldi per le malattie del torace* 2010; 73: 130-134. 2011/01/11. DOI: 10.4081/monaldi.2010.297.
12. Kruis AL, Smidt N, Assendelft WJ, et al. Integrated disease management interventions for patients with chronic obstructive pulmonary disease. *The Cochrane database of systematic reviews* 2013: Cd009437. 2013/10/11. DOI: 10.1002/14651858.CD009437.pub2.
13. Smith J, Wistow G, Holder H, et al. Evaluating the design and implementation of the whole systems integrated care programme in North West London: why commissioning proved (again) to be the weakest link. *BMC health services research* 2019; 19: 228. DOI: 10.1186/s12913-019-4013-5.
14. Frontier Economics. Enablers and barriers to integrated care and implications for Monitor: a report prepared for Monitor. 2012. London: Frontier Economics https://assets.publishing.service.gov.uk/government/uploads/system/uploads/attachment_data/file/287800/Enablers_and_barriers_to_integrated_care_report_June_2012.pdf (accessed 08/05/2020)
15. McDermott I, Checkland K, Moran V, et al. Achieving integrated care through commissioning of primary care services in the English NHS: a qualitative analysis. *BMJ open* 2019; 9: e027622. 2019/04/04. DOI: 10.1136/bmjopen-2018-027622.
16. Sandelowski M. Sample size in qualitative research. Research in Nursing & Health,1995; 18: 179-183. DOI: doi:10.1002/nur.4770180211.
17. Braun V and Clarke V. Using thematic analysis in psychology. *Qualitative Research in Psychology* 2006; 3: 77-101. DOI: 10.1191/1478088706qp063oa.
18. Scally G and Donaldson LJ. The NHS's 50 anniversary. Clinical governance and the drive for quality improvement in the new NHS in England. *BMJ* 1998; 317: 61-65. DOI: 10.1136/bmj.317.7150.61.
19. Pearson C and Watson N. Implementing health and social care integration in Scotland: Renegotiating new partnerships in changing cultures of care. 2018; 26: e396-e403. DOI: 10.1111/hsc.12537.



For peer review only - http://bmjopen.bmj.com/site/about/guidelines.xhtml

20. Rees G, Huby G, McDade L, et al. Joint working in community mental health teams: implementation of an integrated care pathway. 2004; 12: 527-536. DOI: 10.1111/j.1365-2524.2004.00523.x.
21. Cook A, Mulherin M and Seditas K. Partnership working across UK public services. Edinburgh: What works Scotland. 2015. http://whatworksscotland.ac.uk/wp-content/uploads/2015/12/WWS-Evidence-Review-Partnership-03-Dec-2015-.pdf (accessed 08/05/2020)
22. Cumming J. Integrated care in New Zealand. *International journal of integrated care* 2011; 11: e138-e138. 2011/11/18. DOI: 10.5334/ijic.678.
23. Addicott R. Commissioning and contracting for integrated care 2014. The Kings Fund https://www.kingsfund.org.uk/sites/default/files/kings-fund-commissioning-contracting-integrated-care-nov14.pdf (accessed 08/05/2020)
24. Hudson B. Inter-professionality and partnership: an obstacle to integrated care? An explanatory study of professional preferences. Nuffield Institute for Health, 2001.
25. Timmins N and Ham C. The quest for integrated health and social care. A case study in Canterbury, New Zealand. 2013. London: The Kings Fund https://www.kingsfund.org.uk/sites/default/files/field/field_publication_file/quest-integrated-care-new-zealand-timmins-ham-sept13.pdf (accessed 08/05/2020)
26. Best A, Greenhalgh T, Lewis S, et al. Large-system transformation in health care: a realist review. *The Milbank quarterly* 2012; 90: 421-456. 2012/09/19. DOI: 10.1111/j.1468-0009.2012.00670.x.

## FOOTNOTES

### Acknowledgements

The authors would like to thank all the participants who took part in the study. We thank Liz Hill from NIHR ARC West for project support and management during the study. We also thank Martha Elwenspoek and Tim Jones who undertook quantitative research alongside this study and who provided valuable insight and commented on early drafts of this paper.

### Authors' contributions

JB, JD and TS developed and designed the study. JB and TS collected and analysed the data. JB, TS and JD contributed to the write up and completion of the paper.

### Funding

This research was funded by the National Institute for Health Research, Collaboration for Leadership in Applied Health Research and Care West (NIHR CLAHRC West). The views expressed in this article are those of the authors and not necessarily those of the NIHR or the Department of Health and Social Care.

### Conflict of interest

James Dodd was employed as a consultant at the secondary care lung clinic that participated in the research. James Dodd did not analyse the qualitative data or have direct access to the data but did contribute to the interpretation of findings and the write up of the paper. Jonathan Banks and Tracey Stone declare no conflict of interest.



For peer review only - http://bmjopen.bmj.com/site/about/guidelines.xhtml

**Data sharing**
The study's data is drawn from a relatively small number of participants from one NHS Trust Department, a smaller community provider unit and members of the clinical commissioning group. The data features a lot of contextual information which is likely to lead to the identification of individuals. Therefore, to protect the participants' anonymity, the study data will not be made available.

**Ethics approval and consent to participate**
This project received ethical approval from the University of Bristol's Health Sciences Faculty Research Ethics Committee (application ID, 69093). All participants provided informed consent to be interviewed and audio recorded.

For peer review only

# Supplementary file 1 - Evaluation of the Integrated Respiratory Services (IRS) Interview Topic guide V2 22/06/2018

**Part A: Introduction, consent and background**

- Thanks, introduce self, re-state purpose of the interview (to understand the development, management and ongoing use of the integrated respiratory pathway (IRS))
- Discussion of how interview will be recorded, right to withdrawal, issues of confidentiality, anonymisation and informed consent (*face-to-face written consent, telephone verbal consent).* Verbal consent: *switch audio recorder on* - For the audio recording, can I check that:
    - You have read and understood the study information sheet?
    - You know that taking part in the interview is voluntary and you are free to stop the interview at any point and you may skip questions you would prefer not to answer?
    - You agree to our conversation being audio recorded?
    - You understand that quotes from the interview may be used to illustrate our findings but it will not be possible to trace who said them?

**Part B: Working within the integrated research pathway**

- Can you tell us about your role in relation to the integrated respiratory pathway (IRS) and which of the collaborating organisations you work with?
    - Tell us about your experiences of working as part of the IRS
    - How does it differ from the previous pathway of care for respiratory patients
    - What benefits does it offer patients
    - What benefits does it offer for the staff working within the pathway
    - Are there any challenges in working within the pathway
    - Do you perceive any downsides or disadvantages of the pathway for patients
    - Do you perceive any downsides or disadvantages of the pathway for yourself and/or for other members of staff
- The pathway is a collaboration between two different organisations -
    - Tell us about your experience of collaborating and working with the either of these organisations
    - Describe how the relationship has evolved over time
    - What has been the challenges of working with community provider/hospital?
    - What has been the benefits of working with community provider/hospital?
    - Have you trained members of staff at community provider/hospital?
    - Have you received training from members of staff at community provider/hospital?

**Part C: Setting up and managing the pathway**

- Can you tell us about your role in setting up the pathway
    - Were you involved in the design or establishment of the pathway?
    - What prompted the establishment of the pathway?
    - Can you describe the management and governance structures of the pathway and your role in these?
    - What have been the challenges in setting up and managing the pathway?
    - How have these challenges been overcome?
    - What have been the benefits of managing the pathway (as described)?

**Part D: Summary questions**

- What do you see as the future for the IRS, how can it be improved are there areas that are problematic?
- Any questions or issues about the IRS that we have not covered
- Thank interviewee for their time and make sure they have our contact details


For peer review only - http://bmjopen.bmj.com/site/about/guidelines.xhtml

**Supplementary file 2 - COREQ Statement**

# Integrating care between an NHS hospital, a community provider and the role of commissioning: the experience of developing an integrated respiratory service

**Domain 1: Research team and reflexivity**

*Personal Characteristics*

1. Interviewer/facilitator. Which author/s conducted the interview or focus group?
*Jon Banks (JB)*
*Tracey Stone (TS)*

2. Credentials. What were the researcher's credentials? E.g. PhD, MD.
JB*:* PhD
TS: PhD

3. Occupation. What was their occupation at the time of the study?
*JB: Research Fellow*
*TS: Research Associate*

4. Gender. Was the researcher male or female?
*TS - Female.*
*JB - Male*

5. Experience and training. What experience or training did the researcher have?
JB: *Post graduate diploma in social science research methods as part of PhD, experience of collecting and analysing qualitative data with 17 years as a research associate and research fellow.*

*TS: PhD in Research Ethics, 5 years (local and regional) Research Ethics Committee service, Extensive training in qualitative research methods (PhD and subsequent courses) and 15 years' experience conducting qualitative research.*

*Relationship with participants*

6. Relationship established. Was a relationship established prior to study commencement?
*No direct relationship was established with the research participants prior to recruitment.*

7. Participant knowledge of the interviewer. What did the participants know about the researcher? e.g. personal goals, reasons for doing the research?
*The personal goals of the researchers were to complete the aims and objectives of the study only. They were introduced to potential participants by email from the Consultant service lead in the hospital, also co-PI on the project, including full Participant Information which emphasised that taking part in an interview was voluntary. Likewise, in the community setting, the service lead agreed to distribute information to staff. The researchers were not known to the participants prior to the study interviews. When undertaking interviews*

For peer review only

*researchers gave full details about the research along with printed study materials before gaining informed consent. The researchers made their academic standpoint clear to interviewees so they were aware that researchers were from an independent research body not linked to their employers.*

8. Interviewer characteristics. What characteristics were reported about the interviewer/facilitator? e.g. Bias, assumptions, reasons and interests in the research topic.
*The interviewers are social scientists with many years' experience conducting qualitative research on a range of health topics. This research topic was proposed by Academic Clinical collaborators, and not selected by the interviewers due to any particular prior interests or goals related to this research topic.*

**Domain 2: study design**
*Theoretical framework*
9. Methodological orientation and Theory. What methodological orientation was stated to underpin the study? e.g. grounded theory, discourse analysis, ethnography, phenomenology, content analysis
*In the Methods section we explain how we used semi-structured interviews using thematic analysis with NVivo11 software, this was informed by grounded theory and the constant comparison method.*

*Participant selection*
10. Sampling. How were participants selected? e.g. purposive, convenience, consecutive, snowball.
*Purposive sampling, when clinically convenient, was used to select interviewees in order to attempt to capture maximum variation in views and experiences in order that they adequately reflect those of a range of staff involved in the development, implementation and on-going work in the Integrated Respiratory Service.*

11. Method of approach. How were participants approached? e.g. face-to-face, telephone, mail, email
*Please see item 7 above; moreover, potential participants responded to the initial information from the service leads in both organisations by contacting the researchers directly to discuss the project and/or arrange an interview date and time. This took place at or near the participants' places of work for their convenience. All interviewees gave written informed consent.*

12. Sample size. How many participants were in the study?
*We interviewed 19 members of staff, 8 from the secondary care hospital, 9 from the community provider and 2 who were members of the clinical commissioning group.*

13. Non-participation. How many people refused to participate or dropped out? Reasons
*Only one potential participant was eventually not interviewed. Whilst she was willing, after 3 attempts to meet, clinical duties and shift patterns eventually prevented this. This took place towards the end of the study when data saturation was occurring but other interviews*

For peer review only

*were still taking place. For this reason, as well as to avoid inappropriate pressure on the participant the interview did not take place.*

14. Setting of data collection. Where was the data collected? e.g. home, clinic, workplace
*Data collection took place at or near the participants' place of work, at their preference and convenience.*

15. Presence of non-participants. Was anyone else present besides the participants and researchers?
*These were one to one face to face interviews held in private or in quiet public places, (local café in the community and staff social area which was large enough to ensure privacy and where the participants felt comfortable to express their opinions).*

16. Description of sample. What are the important characteristics of the sample? e.g. demographic data, date.
*All the staff interviewed were involved in the development, implementation and on-going operation of the Integrated Respiratory Service. Interview participants were purposively sampled and recruited to cover the range of staff who worked with the service. (See table 1 in paper)*

*Data collection*
17. Interview guide. Were questions, prompts, guides provided by the authors? Was it pilot tested?
*A topic guide was used for all interviews (provided as an additional file). The focus of the topic guide was participants' views and experiences of the Integrated Respiratory Service and was modified as data collection progressed. The topic guide was used flexibly so that interviewees could raise other issues they felt were important whilst also allowing in-depth exploration of emergent themes.*

18. Repeat interviews.
*We did not conduct repeat interviews*

19. Audio/visual recording. Did the research use audio or visual recording to collect the data?
*Yes, all interviews were audio recorded, with the informed consent of participants.*

20. Field notes. Were field notes made during and/or after the interview or focus group?
*Field notes were not made during this study. Reflexive notes were made on password protected computers to aid the iterative development of the topic guide.*

21. Duration. What was the duration of the interviews or focus group?
*Between 14 and 86 minutes*

22. Data saturation. Was data saturation discussed?
*Yes, this was discussed in team meetings.*

For peer review only

23. Transcripts returned. Were transcripts returned to participants for comment and/or correction?
*No.*

**Domain 3: analysis and findings**
*Data analysis*
24. Number of data coders. How many data coders coded the data?
*Two. Data was coded by the researcher Tracey Stone, and Jon Banks, a senior qualitative research fellow. The coding frame was developed by JB and TS following the initial double coding of four interviews covering a range of staff. Coding and iterative developments were discussed at regular meetings.*

25. Description of the coding tree. Did authors provide a description of the coding tree?
*No.*

26. Derivation of themes. Were themes identified in advance or derived from the data?
*Themes were derived deductively from our research questions and inductively from the data.*

27. Software. What software, if applicable, was used to manage the data?
*We used NVivo 11 qualitative software package to manage the data.*

28. Participant checking. Did participants provide feedback on the findings?
*No.*

29. Quotations presented. Were participant quotations presented to illustrate the themes / findings? Was each quotation identified? e.g. participant number
*Yes, with quotations identified using alphanumeric labels for the site, staff role and interview number. These categories are intentionally quite broad. Whilst readers may appreciate more specific information for context, the sample size and variation of roles within the sample in both organisations, would undermine anonymity.*

30. Data and findings consistent. Was there consistency between the data presented and the findings?
*Yes.*

31. Clarity of major themes. Were major themes clearly presented in the findings?
*Yes.*

32. Clarity of minor themes. Is there a description of diverse cases or discussion of minor themes?
*Yes.*

For peer review only

# Integrating care between an NHS hospital, a community provider and the role of commissioning: the experience of developing an integrated respiratory service


| | |
|---|---|
| Journal: | *BMJ Open* |
| Manuscript ID | bmjopen-2020-040267.R1 |
| Article Type: | Original research |
| Date Submitted by the Author: | 27-Aug-2020 |


| | |
|---|---|
| Complete List of Authors: | Banks, Jonathan; NIHR Applied Research Collaboration West, University Hospitals Bristol NHS Foundation Trust ; University of Bristol, School of Population Health Sciences, Bristol Medical School<br>Stone, Tracey; NIHR Applied Research Collaboration West, University Hospitals Bristol ; University of Bristol, School of Population Health Sciences, Bristol Medical School<br>Dodd, James; University of Bristol, Academic respiratory unit, Southmead Hospital |


| | |
|---|---|
| <b>Primary Subject Heading</b>: | Health services research |
| Secondary Subject Heading: | Respiratory medicine, Qualitative research, Health policy |
| Keywords: | Organisation of health services < HEALTH SERVICES ADMINISTRATION & MANAGEMENT, QUALITATIVE RESEARCH, Adult thoracic medicine < THORACIC MEDICINE, Health policy < HEALTH SERVICES ADMINISTRATION & MANAGEMENT |
| | |


SCHOLARONE™
Manuscripts

*I, the Submitting Author has the right to grant and does grant on behalf of all authors of the Work (as defined in the below author licence), an exclusive licence and/or a non-exclusive licence for contributions from authors who are: i) UK Crown employees; ii) where BMJ has agreed a CC-BY licence shall apply, and/or iii) in accordance with the terms applicable for US Federal Government officers or employees acting as part of their official duties; on a worldwide, perpetual, irrevocable, royalty-free basis to BMJ Publishing Group Ltd ("BMJ") its licensees and where the relevant Journal is co-owned by BMJ to the co-owners of the Journal, to publish the Work in this journal and any other BMJ products and to exploit all rights, as set out in our licence.*

*The Submitting Author accepts and understands that any supply made under these terms is made by BMJ to the Submitting Author unless you are acting as an employee on behalf of your employer or a postgraduate student of an affiliated institution which is paying any applicable article publishing charge ("APC") for Open Access articles. Where the Submitting Author wishes to make the Work available on an Open Access basis (and intends to pay the relevant APC), the terms of reuse of such Open Access shall be governed by a Creative Commons licence – details of these licences and which Creative Commons licence will apply to this Work are set out in our licence referred to above.*

*Other than as permitted in any relevant BMJ Author's Self Archiving Policies, I confirm this Work has not been accepted for publication elsewhere, is not being considered for publication elsewhere and does not duplicate material already published. I confirm all authors consent to publication of this Work and authorise the granting of this licence.*

For peer review only

# Integrating care between an NHS hospital, a community provider and the role of commissioning: the experience of developing an integrated respiratory service

**Authors**
Jon Banks[1,2] https://orcid.org/0000-0002-3889-6098
Tracey Stone[1,2] https://orcid.org/0000-0003-2627-3843
James W Dodd[3] https://orcid.org/0000-0003-4805-5759

**Affiliations**
1. The National Institute for Health Research, Applied Research Collaboration West (NIHR ARC West), University Hospitals Bristol NHS Foundation Trust, BS1 2NT Bristol UK
2. Population Health Sciences, Bristol Medical School, University of Bristol, UK BS8 1UD
3. Academic respiratory unit, University of Bristol, Southmead Hospital Bristol, UK BS10 5NB

**Corresponding author**
Jon Banks
9th Floor, Whitefriars, Lewins Mead,
Bristol, United Kingdom BS1 2NT
Email: Jon.Banks@Bristol.ac.uk
Tel: 0117 342 1244

**Word count**
4059

**Key Words**
Delivery of health care, integrated; qualitative research; pulmonary disease, chronic obstructive

## Abstract

### Objectives
An integrated respiratory service was commissioned in 2016 in a UK region to support patients with chronic obstructive pulmonary disease. The service brought together the respiratory department of a National Health Service hospital and a not-for-profit community provider. This paper evaluates: (a) the perceived efficacy of integrated working between the organisations from the perspective of staff; and (b) the relationship between commissioning and integration of the services.



For peer review only - http://bmjopen.bmj.com/site/about/guidelines.xhtml

## Design

Semi-structured interviews with staff from the three organisations involved in the integrated respiratory service. Staff were purposefully sampled. The interviews were audio recorded, transcribed and analysed thematically.

## Setting

Secondary care respiratory unit; community provider of respiratory care; and a clinical commissioning group.

## Participants

Nineteen interview participants: nine from the community provider; eight from the hospital; and two from the clinical commissioning group.

## Results

Staff identified lack of integration between the organisations characterised by: poor communication, lack of trust, absence of shared information technology, and ineffective integrative initiatives. The commissioning process created barriers to integration including: contractual limitations which prevented pathway development, absence of agreed clinical governance arrangements, and lack of recognition of community work undertaken by hospital staff. Positive working relationships were established over time as staff recognised the skills that each had to offer.

## Conclusions

The commissioning process underpinned the relationship between the organisations and contributed to distrust and negative perceptions of the 'other'. Commissioning an integrated service should incorporate dialogue with stakeholders as early as possible and before the contract is finalised to develop a bedrock of trust.


For peer review only

## Abbreviations

CCG: Clinical Commissioning Group
CSU: Commissioning Support Unit (organisations which support the CCGs in commissioning services)
COPD Chronic obstructive pulmonary disease
COREQ: Guidelines: Consolidated criteria for reporting qualitative research
HOT: Hospital Outpatient Treatment clinic
ICPs: Integrated care pathways
IRS: Integrated Respiratory Service

## Strengths and limitations of this study

- In depth interviews with staff from a community health care provider, a hospital and the commissioning organisation highlighted barriers and facilitators to establishing an integrated care pathway.
- A range of clinical and non-clinical participants were recruited from organisations involved in the formation and delivery of an integrated respiratory care pathway.
- Interview data reflect a particular time period in the development of the integrated respiratory pathway but the relationship between the organisations was dynamic and evolving.
- The study gives an in-depth account of the experiences of developing and delivering one integrated care service, the findings may not be transferable to other settings.
- The study focuses on staff experiences and does not offer data on patient experiences of the integrated care respiratory pathway.

## Introduction

Integrated care pathways (ICPs) have increasingly been used in healthcare to bring together different organisations associated with the care and treatment of a patient population. ICPs link secondary and primary care and/or organisations from sectors such as social care. The rationale is that integrated care can be '*the best possible care for the patient, delivered by the most suitable health professional, at the optimal time, in the most suitable setting.*'[1] It also offers the potential for efficiency savings, freeing up clinical resources from hospitals



For peer review only - http://bmjopen.bmj.com/site/about/guidelines.xhtml

and other health care sites. This development of ICPs is central to the UK National Health Service Long Term Plan.[2 3]

An integrated respiratory service (IRS) was commissioned by a clinical commissioning group (CCG) in 2016 in a region of the UK to support patients with chronic obstructive pulmonary disease (COPD). We have not named the region to preserve participant anonymity. The service brought together the respiratory department of a secondary care hospital and a not for profit community provider. The rationale of the development was that care could be shared between secondary care and the community to the benefit of patients, who could receive care and support in the community, at the same time reducing demand on secondary care. The development of integrated care pathways for respiratory care is supported by The British Thoracic Society.[1]

The new service commissioned a community provider to deliver four respiratory services: oxygen assessment; pulmonary rehabilitation; hospital admission avoidance; and early supported discharge from hospital. The service was integrated in the sense that patients moved between the two organisations at different points of the care pathways. The main points of crossover were joint triaging of referrals for oxygen assessment and pulmonary rehabilitation to assess which patients were appropriate for community care; and phone consultations between the community and the Hospital Outpatient Treatment (HOT) clinic. The HOT clinic was a long-established admission avoidance service used by primary care. It was staffed by a specialist respiratory nurse and consultant respiratory physician. In relation to the new service it was the main point of contact for community staff who needed clinical advice and support.



For peer review only - http://bmjopen.bmj.com/site/about/guidelines.xhtml

A board was established to guide the development of the service at a strategic level and was made up of the commissioning team along with senior clinical and managerial leads from both organisations. There were also joint meetings between clinical staff on a quarterly basis to: forge interworking relationships between staff; and disseminate knowledge and training.

Previous research in relation to integrated respiratory care has identified some promising results in terms of integration and admission avoidance[4-6] but in general the results have been mixed.[7-9] Despite the policy prioritisation associated with integrated care, research and evaluations have not produced clear evidence of effectiveness.[3 10-14] Studies have shown how tensions between the professional cultures of different organisations can impede integration.[15 16] The commissioning process (the contractual and clinical governance arrangements that underpin collaboration) has also been identified as shaping the working relationships and quality of integration between organisations involved in care pathways.[17] We undertook a qualitative evaluation of the IRS to understand: (a) the extent to which staff within the IRS worked together to provide an integrated care pathway; and (b) the relationship between commissioning and the development of an integrated service.

## Patient and public involvement

This study looked at the process of integrating care from the perspective of staff working for the care providers and commissioning organisation. Because of the staff focus we decided that patient and public involvement was not appropriate.



For peer review only - http://bmjopen.bmj.com/site/about/guidelines.xhtml

## Methods

The study took place between May 2018 and May 2019 and used semi-structured interviews with participants purposively sampled from the three organisations involved: the secondary care hospital, the community service provider and CCG commissioners. Inclusion criteria were staff who were involved in the formation and delivery of the IRS.

In the hospital and community organisation, study information was distributed by senior staff and potential participants were invited to contact the research team. A reminder email was sent after 2 weeks. Sampling sought to capture the range of roles in the IRS including: senior clinical/management staff, team leads, clinical and support staff. The research team contacted those involved with the commissioning process directly by email and sent them research information inviting them to respond.

All participants gave informed consent. Interviews were conducted by JB and TS and were audio-recorded. A topic guide was developed by the research team to support the interviews (supplementary file 1) based on the study research questions and issues raised by relevant research literature. The guide was used flexibly and adjusted iteratively as interviews progressed. Data were reviewed throughout the data collection period at research team meetings until we were satisfied that we had sampled relevant staff from both organisations and that no new data were emerging.[18]

Audio recordings of interviews were transcribed by a professional transcribing service who entered into a confidentiality agreement with the research team and were anonymised and imported into NVivo11. We used a data driven inductive approach to identify patterns and themes across the dataset and by research site. The data were analysed thematically.[19] Analysis commenced with open coding. JB and TS each coded a sample of early transcripts

For peer review only

and jointly developed an initial coding framework. Following a further round of double coding, the framework was agreed and applied to the full dataset by TS. The analysis proceeded by developing broader categories through comparison across the transcripts and identifying higher-level recurring themes. Members of the study team met regularly to discuss analysis.

The research was approved by a UK University Research Ethics Committee and the Health Research Authority in England. It adhered to the COREQ guidelines for qualitative research (supplementary file 2).

## Results

Nineteen interviews were conducted between October and December 2018, approximately 2 years after the IRS started. Seventeen were face-to-face and two were over the telephone. Interviews lasted between 14-86 minutes. Details of participants by role are detailed in table 1.

## Table 1: participant details

| **Sector / professional background** | **Participants** |
|---|---|
| ***Hospital*** | |
| Respiratory nurse | 3 |
| Respiratory physiotherapist | 2 |
| Respiratory physiotherapist support | 1 |
| Respiratory team leads | 2 |
| ***Community Provider*** | |
| Respiratory nurse | 2 |
| Respiratory physiotherapists | 2 |
| Nursing support staff | 3 |
| Service lead/management | 2 |
| ***Clinical Commissioning Group*** | |
| Commissioners | 2 |
| **Total participants** | **19** |



For peer review only - http://bmjopen.bmj.com/site/about/guidelines.xhtml

| Notes on sectors | |
|---|---|
| *Hospital* | NHS Hospital Trust Respiratory department (teaching hospital). Included nursing staff, physiotherapists, team leads and consultants. |
| *Community provider* | Participants providing/leading nursing services delivered in patients' homes, primary care and community settings. Included nursing staff, nursing support staff, physiotherapists and team leads. |
| *Clinical Commissioning Group* | Participants were key drivers in the development of the Integrated Respiratory Service (IRS) and were pivotal figures in establishing the relationships between secondary care and the community provider. |

For peer review only

We present our findings under two broad themes: whether integration is working; and the impact of commissioning on integration. Quotes are tagged by study ID and organisation. Participant roles are not included to preserve anonymity.

## Integration – is it working?

In this section we look at the working relationship between staff from both organisations to assess the extent to which they were able to work together effectively to deliver an integrated service to patients.

### *It's a good idea but …*

Participants from both organisations thought the IRS was a good idea and felt the service offered advantages for patients. The pulmonary rehabilitation service offered a choice of location for the six-week course at either the established hospital service or a new programme located at a sports centre. Staff from both organisations supported patient access to a specialist community team to avoid hospital admission and the assessment of patient oxygen in community settings,

> *I think the concept of an integrated respiratory team working across primary and secondary care is a great idea but I think there has to be that shift into community care and keeping these patients out of hospital and we have to change the way that we're thinking about managing these patients.*
> ***(02- hospital)***



For peer review only - http://bmjopen.bmj.com/site/about/guidelines.xhtml

> *I think it's a good thing. I just think we all need to be consistent in what we do and how we provide it really.* **(11-community)**

Despite this positive perspective, many staff felt that integration had not developed as anticipated, especially joint working relationships between staff from both organisations,

> *I don't think we are integrated at all.* **(14-community)**

> *There has to be a responsibility for the patient but because we're integrated, they should be able to pass between the two providers seamlessly and sometimes that doesn't happen.* **(04-hospital)**

## *Barriers to integration – communication, trust and perception of the 'other'*

The lack of integration was evident in the way that the 'other' organisation was viewed. There was a perception of imbalance and a lack of reciprocity. The communication between the organisations became blocked and misaligned as these perceptions affected their inter-organisational relationships. The hospital staff generally felt that their experience and expertise was not being utilised by the community team,

> *We've always said right from the start, "If you've ever got any questions or concerns phone us and we'll do our best to help you." That very rarely ever happens, even now it hardly happens.* **(07-hospital)**

Despite this, community staff did not feel encouraged to ask for help,

> *You can ring a hot consultant but they're always madly busy because they've got a hot clinic. Along with that is the irritation. We're an irritation.* **(13-Community)**

The perception of imbalance came through in comments from both organisations whereby 'they' had obligations or were undertaking actions that the 'other' did not reciprocate,

> *We refer to them, but in the duration of the prevention of hospital admission services running – we've only had three referrals from them …* **(08-community)**

> *If we pick the patients up who are under the team in the community, when they're in hospital, they would never phone us and say, "We've sent in Mrs Bloggs today, can you catch up with her?" There isn't that sort of communication. They like us to let them know when we've caught up with a patient, but they don't do it reciprocally.* **(07-hosptital)**

There was a sense in the early stages of the service that patients were appropriated as a currency through which either organisation could secure their position or legitimacy. The



For peer review only - http://bmjopen.bmj.com/site/about/guidelines.xhtml

community organisations perceived that hospital staff dominated the mechanisms of joint triage which gave them control over patient care, whereas hospital staff were concerned that the community organisation would take their patients,

> *[The Hospital] seem to have a lot of control, they get all the referrals, so we don't get sight of the referrals they get so it's like a centralised referral system but they manage it so it doesn't feel very joint and there's quite a few processes that are like that with [the Hospital].* ***(17-community)***

> *To start with it just felt, and we were informed that they were being told by senior members of the team to take everybody, any referral had to go to [the community team]. It just felt that when they were set up all they wanted to do – the [community] team just wanted to take all the patients from us.* ***(07-hospital)***

Members of the community team described the relationship between the organisations in hierarchical terms. At times, they felt they were only able to undertake key aspects of their work with the approval of the hospital team. On the other, hand the hospital team described a feeling of uncertainty about the clinical skills held by the community team and the degree to which they could provide clinically appropriate patient care,

> *We maintain a professional relationship. We have good conversations with them. A couple of them are a bit patronising. I just let it go over my head and I counsel those that don't find that very easy, but I suppose my gut feeling is that we all feel a bit substandard and that's not an integrated team.* ***(13-community)***

> *The general feeling is that the skills in the community team are not as high as the skills and experience within the team sitting in the hospital.* ***(02-hospital)***

The perceived skill differential also manifested itself in a lack of trust and confidence in each other and each other's organisation,

> *I think one of the biggest barriers to start with was change – change within our team and a change of mindset and actually these patients can be managed in the community* ***(02-hospital)***

> *The final pathway we managed to get up and running was the prevention of admission and we kept going round and round in circles with it and primarily because I think that the team in the hospital didn't think we had the expertise.* ***(18-community)***



For peer review only - http://bmjopen.bmj.com/site/about/guidelines.xhtml

### *IT systems*

Lack of access to a shared patient record system was consistently cited by staff in both organisations as a barrier to integration. A number of workarounds were developed which included community staff being able to log on to the secondary care system but this entailed manually transposing content onto their system which proved to be cumbersome and duplicated community work. The lack of a shared system served to maintain distance between the organisations,

> *Here, we have access to a computer system and we put a lot of information on there about our patients when we've seen them. For early supported discharge, for the respiratory nurse clinics, and to do with oxygen. Whilst the [community] team didn't have access – not all of them had access to that system … So, straight away, we couldn't see what they were doing, and they couldn't see what we were doing.* (**01-hospital)**

> *The main barrier is the fact we use different note systems. It's a huge barrier. – either they've got read only access but not on all their computers in the hospital so it's not accessible for them. We use the same systems as the GPs, district nurses, community matrons which has a plethora of information that if when a patient is admitted into hospital they get opened up, it would give them a lot more awareness of what we've been trying to do with that patient.* ***(11-community)***

### *Integrative mechanisms*

There were differing perspectives on the joint staff meetings. The hospital team were more positive whereas community members did not feel that these meetings engaged with the barriers to integrated working,

> *We have these meetings every three or four months with this team and every time before we go I wish we could have an open and honest conversation about how people feel, but nobody's ever brave enough to do it, so I'd rather all cards on the table … there have been a few difficult conversations, few snipey remarks.* ***(13-community)***

> *We've had good meetings where we've talked about maybe issues that we've both experienced and we're maybe prescribing oxygen or you know, certain patients – what do we do about this particular patient you know and that's been really useful for both sides I think but yes it's good to have those regular meet ups I think and just discuss the things we come across on a daily basis.* ***(03-hospital)***



For peer review only - http://bmjopen.bmj.com/site/about/guidelines.xhtml

### *Integrated working – what worked?*

Where working relationships did improve this often came about informally. Some nurses were proactive about phoning and emailing colleagues from their partner organisation in cases of uncertainty. More importantly, as staff worked together on joint triage and phone consultations their knowledge of each other and the complimentary skills and experience they brought to the pathway grew, along with developing mutual respect. It was these contacts rather than the more formalised group meetings that enabled a more effective and comfortable level of communication,

> *Over time obviously the service in secondary care has become more trusting of what we can deliver in the community and trust the fact that if we've got any concerns, then we will report those in to the consultant and if necessary, we'll either arrange for somebody to go to a hot clinic appointment because we can access that by contacting secondary care direct or we will admit.* ***(18-community)***

> *[With] time comes experience and now we're starting to feel more comfortable with it and we feel that when we have a conversation with, well personally if I'm having a conversation with [the community team] I feel more – you know the conversations are obviously coming from people who've got experience now and I think that improves with time really.* ***(04-hospital)***

## The commissioning process and integrated working

In this section we look at the role of the commissioning process to provide context to issues highlighted above.

### *A 'new' community service?*

The services that the community organisation were commissioned to provide were not completely new. The hospital had been providing similar services, some of them in community settings, prior to the IRS. Pulmonary rehab was run at a community hospital site. There was also an established 'early supported discharge' programme involving hospital-based specialist nursing and physiotherapists providing clinical support for patients at home. The commissioning of services already being undertaken by the hospital therefore created confusion, resentment and uncertainty, for both healthcare providers,



For peer review only - http://bmjopen.bmj.com/site/about/guidelines.xhtml

*Once we started to recruit into the service we then started to find that [the hospital) were saying well why are you recruiting to that when we've got that already? So that's kind of where the…certain amount of confusion started.* ***(18-community)***

*We were already out in the community and providing oxygen, providing pulmonary rehabilitation and we were providing early supported discharge and then all of a sudden we had a team that were then commissioned to provide the same services.* ***(02-hospital)***

This established a foundation of mistrust between the two organisations – the hospital saw the community work they had established being removed from them whereas the community organisation felt the hospital were holding on to sections of the service the community had been commissioned to undertake. Pathways developed and integrated structures were created but the weight of unresolved tensions continued to undermine integration,

*I think we all went in with a bit of you know, well we need to protect our end of things.* ***(18-community)***

*The way it was set up probably wasn't the most ideal. We originally sat down and we informed commissioners of what we provided but I don't think that was taken on board at all because when the original business case then came out, saying that this is actually what they wanted [the community team] to provide, was actually everything that we were providing so to start with we'd already kind of got our heckles up a little bit about that process* ***(02-hospital)***

## *Commissioning anomalies*

The 'early supported discharge pathway' element of the new service never started. The contract with the community provider funded care five days a week whereas the hospital delivered care seven days a week which was aligned with the hospital's early supported discharge pathway. The outcome was that the secondary care hospital remained as provider of early supported discharge work,

*It just impacts on the amount of work we can give them as far as an admission avoidance or an early supported discharge pathway was concerned. Because we couldn't give them a patient on a Thursday because they wouldn't be able to see them over the weekend, they'd be handing them back for us to look after.* ***(06-hospital)***



For peer review only - http://bmjopen.bmj.com/site/about/guidelines.xhtml

This limitation served to widen the gulf between the organisations. For the hospital team it fed into a view that the community organisation was not a fully-fledged health care organisation who could meet the service specifications that they were providing. The community team felt restricted by the contract and unable to prove themselves.

### *Commissioning and clinical governance*

Clinical governance,[20] the framework by which the quality of patient care is assured, was a divisive issue between the organisations. The hospital had an NHS clinical resource in the form of established respiratory consultants, nurses and physiotherapists which formed the bedrock of their clinical supervision. The community organisation had to build their team around their nursing and physiotherapist staff. They did not have the same access to clinical supervision, particularly consultant support. The solution was for community staff to phone HOT clinic clinicians for advice and support when they required it. There was no dedicated time provided for this and the HOT clinic clinicians felt it placed more demand on their time and added a level of uncertainty to their clinical responsibilities,

> *We get a lot of telephone calls when we are doing our HOT weeks, being asked questions that are really basic… It's one of the weaknesses I think in this process is [the community team] does not have in place clinical governance arrangements that we would find acceptable* ***(19-hospital)***
>
> *When we were discussing the service spec with the CCG and the CSU [commissioning support unit] for the service here, we raised the fact that it would need to have an element of clinical supervision from the consultants built into the spec … They didn't want to allocate any money to that* ***(18-community)***

## Discussion

### Summary

The IRS was developed to incorporate aspects of specialist respiratory care into community settings. To work effectively, the new service required close working between staff from the hospital and community providers. This involved joint triage sessions and clinical dialogue between staff. Our study looked at integration from the perspective of staff with a focus on



For peer review only - http://bmjopen.bmj.com/site/about/guidelines.xhtml

joint working practices. Our results indicate that effective integration between the organisations had not been achieved. There were substantive problems generated by IT incompatibility but more significant was the level of mistrust that staff felt toward their partner organisation which undermined effective joint working. Negative perceptions of power, hierarchy and control were expressed by staff from both organisations. However, it was notable how the relationships between organisations in the IRS improved over time. The barriers of mistrust gave way to a growing recognition of the knowledge and skills held by all staff. Interaction became easier, leading to something closer to integrated working.

## Comparison with other literature

There are few qualitative studies of integrated care in relation to respiratory disease[21 22] and we are not aware of studies that focus on the processes of integration and commissioning. As such, we have situated our findings in the wider literature around integrated care and commissioning. Our findings resonate with other studies [23 24] which show support for the principle of an integrated service among staff but with limited evidence that it had been achieved. Staff in the IRS felt there was a hierarchical relationship between organisations which previous studies have identified as disruptive to integrated working. Similarly, inter-organisational mistrust was identified as the key barrier to a widespread programme of integration in New Zealand.[25]

We explored the role of commissioning in shaping the relationship between the IRS organisations. The framework of integration is aligned with what a Kings Fund report[26] identifies as an 'alliance' model, whereby a number of providers hold contracts with the commissioning body. They argue that the alliance model requires a high level of trust between organisations to work effectively. Also, creating mechanisms and structures that



For peer review only - http://bmjopen.bmj.com/site/about/guidelines.xhtml

bring organisations together does not in itself create effective interprofessional relationships.[27] The joint meetings intended to support integration in the IRS were viewed with a mixture of ambivalence and hostility. Similarly, the contract between organisations should not be seen as the mechanism of integration, '*the contract is merely the 'scaffolding' for the integrated model*.'[26] The Kings Fund argue that the structure of collaborations between health care providers needs to be developed between all parties before the contract is finalised.[26] A similar argument underpinned progress made in New Zealand where they moved away from a top-down approach to one which encouraged collaborations at a grass roots level prior to formal integration as a building block to integrated care.[28] This resonates with recommendations made following the introduction of integrated care systems in London which argues that commissioners need to engage all stakeholders.[15 29] They also highlight the need to attend to history which is an issue that slowed the progress of the service in this study.

## Strengths and limitations

In depth interviews with a range of staff from organisations involved in the IRS enabled understanding of factors that inhibited and facilitated an integrated service. Our findings can support the development of integrated services in other areas but we acknowledge that the data may not be transferable to all settings. The data are taken from a particular time point in the development of the service. Had interviews taken place earlier or later in the process the results may have carried a different emphasis. The study does not evaluate patient outcome and experience. As part of a wider study, a quantitative paper has been submitted for publication elsewhere looking at clinical outcome data. However, further research is needed to capture patient experience of this pathway.



For peer review only - http://bmjopen.bmj.com/site/about/guidelines.xhtml

## Implications for policy

Our results highlight challenges and offer lessons at a time when integrated services are being developed throughout the UK. There was a bedrock of good will around the idea of integration among health care staff in our study. Commissioners of integrated care can build upon such positive orientation through use of pre-contract partnership building so that providers have opportunities to shape and effectively co-commission a new service. It will also create opportunity to: address structural issues like IT; agree clinical governance arrangements; and engender inter-organisational trust.

## References

1. British Thoracic Society. Position statement: integrated respiratory care 2019: British Thoracic Society, 2019.
2. NHS. Long Term Plan 2019.
3. Baxter S, Johnson M, Chambers D, et al. The effects of integrated care: a systematic review of UK and international evidence. *BMC health services research* 2018;18(1):350. doi: 10.1186/s12913-018-3161-3 [published Online First: 2018/05/12]
4. Casas A, Troosters T, Garcia-Aymerich J, et al. Integrated care prevents hospitalisations for exacerbations in COPD patients. *The European respiratory journal* 2006;28(1):123-30. doi: 10.1183/09031936.06.00063205 [published Online First: 2006/04/14]
5. Gillett K, Lippiett K, Astles C, et al. Managing complex respiratory patients in the community: an evaluation of a pilot integrated respiratory care service. 2016;3(1):e000145. doi: 10.1136/bmjresp-2016-000145 %J BMJ Open Respiratory Research
6. Kruis AL, Smidt N, Assendelft WJ, et al. Integrated disease management interventions for patients with chronic obstructive pulmonary disease. *The Cochrane database of systematic reviews* 2013(10):Cd009437. doi: 10.1002/14651858.CD009437.pub2 [published Online First: 2013/10/11]
7. Boland MR, Tsiachristas A, Kruis AL, et al. The health economic impact of disease management programs for COPD: a systematic literature review and meta-analysis. *BMC pulmonary medicine* 2013;13:40. doi: 10.1186/1471-2466-13-40 [published Online First: 2013/07/04]
8. Kruis AL, Boland MRS, Assendelft WJJ, et al. Effectiveness of integrated disease management for primary care chronic obstructive pulmonary disease patients: results of cluster randomised trial. 2014;349:g5392. doi: 10.1136/bmj.g5392 %J BMJ : British Medical Journal
9. Carron T, Bridevaux PO, Lörvall K, et al. Feasibility, acceptability and effectiveness of integrated care for COPD patients: a mixed methods evaluation of a pilot



For peer review only - http://bmjopen.bmj.com/site/about/guidelines.xhtml

community-based programme. *Swiss Med Wkly* 2017;147:w14567. doi: 10.4414/smw.2017.14567 [published Online First: 2017/12/13]
10. Georghiou T, Keeble E. Age UK's Personalised Integrated Care Programme. Evaluation of impact on Hospital Activity, 2019.
11. Georghiou T. Little evidence that integrated care initiatives deliver. Health Services Journal 2019.
12. National Audit Office. Health and social care integration, 2017.
13. Scobie S. Are patients benefitting from better integrated care? QualityWatch blog Nuffield Trust and Health Foundation, 2019.
14. Shepperd S, Doll H, Angus RM, et al. Admission avoidance hospital at home. *The Cochrane database of systematic reviews* 2008(4):Cd007491. doi: 10.1002/14651858.Cd007491 [published Online First: 2008/10/10]
15. Smith J, Wistow G, Holder H, et al. Evaluating the design and implementation of the whole systems integrated care programme in North West London: why commissioning proved (again) to be the weakest link. *BMC health services research* 2019;19(1):228. doi: 10.1186/s12913-019-4013-5
16. Frontier Economics. Enablers and barriers to integrated care and implications for Monitor: a report prepared for Monitor, 2012.
17. McDermott I, Checkland K, Moran V, et al. Achieving integrated care through commissioning of primary care services in the English NHS: a qualitative analysis. *BMJ open* 2019;9(4):e027622. doi: 10.1136/bmjopen-2018-027622 [published Online First: 2019/04/04]
18. Sandelowski M. Sample size in qualitative research. 1995;18(2):179-83. doi: doi:10.1002/nur.4770180211
19. Braun V, Clarke V. Using thematic analysis in psychology. *Qualitative Research in Psychology* 2006;3(2):77-101. doi: 10.1191/1478088706qp063oa
20. Scally G, Donaldson LJ. The NHS's 50 anniversary. Clinical governance and the drive for quality improvement in the new NHS in England. *BMJ* 1998;317(7150):61-65. doi: 10.1136/bmj.317.7150.61
21. Wodskou PM, Høst D, Godtfredsen NS, et al. A qualitative study of integrated care from the perspectives of patients with chronic obstructive pulmonary disease and their relatives. *BMC health services research* 2014;14:471. doi: 10.1186/1472-6963-14-471 [published Online First: 2014/10/04]
22. Waibel S, Vargas I, Aller MB, et al. The performance of integrated health care networks in continuity of care: a qualitative multiple case study of COPD patients. *Int J Integr Care* 2015;15:e029. doi: 10.5334/ijic.1527 [published Online First: 2015/07/28]
23. Pearson C, Watson N. Implementing health and social care integration in Scotland: Renegotiating new partnerships in changing cultures of care. 2018;26(3):e396-e403. doi: 10.1111/hsc.12537
24. Rees G, Huby G, McDade L, et al. Joint working in community mental health teams: implementation of an integrated care pathway. 2004;12(6):527-36. doi: 10.1111/j.1365-2524.2004.00523.x
25. Cumming J. Integrated care in New Zealand. *International journal of integrated care* 2011;11(Spec 10th Anniversary Ed):e138-e38. doi: 10.5334/ijic.678 [published Online First: 2011/11/18]
26. Addicott R. Commissioning and contracting for integrated care The Kings Fund 2014.



For peer review only - http://bmjopen.bmj.com/site/about/guidelines.xhtml

27. Hudson B. Inter-professionality and partnership: an obstacle to integrated care? An explanatory study of professional preferences. Nuffield Portfolio Programme Report No15, 2001.
28. Timmins N, Ham C. The quest for integrated health and social care. A case study in Canterbury, New Zealand. London: The Kings Fund 2013.
29. Best A, Greenhalgh T, Lewis S, et al. Large-system transformation in health care: a realist review. *The Milbank quarterly* 2012;90(3):421-56. doi: 10.1111/j.1468-0009.2012.00670.x [published Online First: 2012/09/19]

## FOOTNOTES

### Acknowledgements

The authors would like to thank all the participants who took part in the study. We thank Liz Hill from NIHR ARC West for project support and management during the study. We also thank Martha Elwenspoek and Tim Jones who undertook quantitative research alongside this study and who provided valuable insight and commented on early drafts of this paper.

### Authors' contributions

JB, JD and TS developed and designed the study. JB and TS collected and analysed the data. JB, TS and JD contributed to the write up and completion of the paper.

### Funding

This research was funded by the National Institute for Health Research, Collaboration for Leadership in Applied Health Research and Care West (NIHR CLAHRC West). The views expressed in this article are those of the authors and not necessarily those of the NIHR or the Department of Health and Social Care. Grant no: NL601187440GB

### Conflict of interest

James Dodd was employed as a consultant at the secondary care lung clinic that participated in the research. James Dodd did not analyse the qualitative data or have direct access to the data but did contribute to the interpretation of findings and the write up of the paper. Jonathan Banks and Tracey Stone declare no conflict of interest.

### Data sharing

The study's data is drawn from a relatively small number of participants from one NHS Trust Department, a smaller community provider unit and members of the clinical commissioning group. The data features a lot of contextual information which is likely to lead to the identification of individuals. Therefore, to protect the participants' anonymity, the study data will not be made available.

### Ethics approval and consent to participate

This project received ethical approval from the University of Bristol's Health Sciences Faculty Research Ethics Committee (application ID, 69093). All participants provided informed consent to be interviewed and audio recorded.

For peer review only

## Supplementary file 1 - Evaluation of the Integrated Respiratory Services (IRS) Interview Topic guide V2 22/06/2018

**Part A: Introduction, consent and background**

- Thanks, introduce self, re-state purpose of the interview (to understand the development, management and ongoing use of the integrated respiratory pathway (IRS))
- Discussion of how interview will be recorded, right to withdrawal, issues of confidentiality, anonymisation and informed consent (*face-to-face written consent, telephone verbal consent).* Verbal consent: *switch audio recorder on* - For the audio recording, can I check that:
  - You have read and understood the study information sheet?
  - You know that taking part in the interview is voluntary and you are free to stop the interview at any point and you may skip questions you would prefer not to answer?
  - You agree to our conversation being audio recorded?
  - You understand that quotes from the interview may be used to illustrate our findings but it will not be possible to trace who said them?

**Part B: Working within the integrated research pathway**

- Can you tell us about your role in relation to the integrated respiratory pathway (IRS) and which of the collaborating organisations you work with?
  - Tell us about your experiences of working as part of the IRS
  - How does it differ from the previous pathway of care for respiratory patients
  - What benefits does it offer patients
  - What benefits does it offer for the staff working within the pathway
  - Are there any challenges in working within the pathway
  - Do you perceive any downsides or disadvantages of the pathway for patients
  - Do you perceive any downsides or disadvantages of the pathway for yourself and/or for other members of staff
- The pathway is a collaboration between two different organisations -
  - Tell us about your experience of collaborating and working with the either of these organisations
  - Describe how the relationship has evolved over time
  - What has been the challenges of working with community provider/hospital?
  - What has been the benefits of working with community provider/hospital?
  - Have you trained members of staff at community provider/hospital?
  - Have you received training from members of staff at community provider/hospital?

**Part C: Setting up and managing the pathway**

- Can you tell us about your role in setting up the pathway
  - Were you involved in the design or establishment of the pathway?
  - What prompted the establishment of the pathway?
  - Can you describe the management and governance structures of the pathway and your role in these?
  - What have been the challenges in setting up and managing the pathway?
  - How have these challenges been overcome?
  - What have been the benefits of managing the pathway (as described)?

**Part D: Summary questions**

- What do you see as the future for the IRS, how can it be improved are there areas that are problematic?
- Any questions or issues about the IRS that we have not covered
- Thank interviewee for their time and make sure they have our contact details


For peer review only - http://bmjopen.bmj.com/site/about/guidelines.xhtml

**Supplementary file 2 - COREQ Statement**

# Integrating care between an NHS hospital, a community provider and the role of commissioning: the experience of developing an integrated respiratory service

**Domain 1: Research team and reflexivity**

*Personal Characteristics*

1. Interviewer/facilitator. Which author/s conducted the interview or focus group?
*Jon Banks (JB)*
*Tracey Stone (TS)*

2. Credentials. What were the researcher's credentials? E.g. PhD, MD.
JB*:* PhD
TS: PhD

3. Occupation. What was their occupation at the time of the study?
*JB: Research Fellow*
*TS: Research Associate*

4. Gender. Was the researcher male or female?
*TS - Female.*
*JB - Male*

5. Experience and training. What experience or training did the researcher have?
JB: *Post graduate diploma in social science research methods as part of PhD, experience of collecting and analysing qualitative data with 17 years as a research associate and research fellow.*

*TS: PhD in Research Ethics, 5 years (local and regional) Research Ethics Committee service, Extensive training in qualitative research methods (PhD and subsequent courses) and 15 years' experience conducting qualitative research.*

*Relationship with participants*

6. Relationship established. Was a relationship established prior to study commencement?
*No direct relationship was established with the research participants prior to recruitment.*

7. Participant knowledge of the interviewer. What did the participants know about the researcher? e.g. personal goals, reasons for doing the research?
*The personal goals of the researchers were to complete the aims and objectives of the study only. They were introduced to potential participants by email from the Consultant service lead in the hospital, also co-PI on the project, including full Participant Information which emphasised that taking part in an interview was voluntary. Likewise, in the community setting, the service lead agreed to distribute information to staff. The researchers were not known to the participants prior to the study interviews. When undertaking interviews*

For peer review only

*researchers gave full details about the research along with printed study materials before gaining informed consent. The researchers made their academic standpoint clear to interviewees so they were aware that researchers were from an independent research body not linked to their employers.*

8. Interviewer characteristics. What characteristics were reported about the interviewer/facilitator? e.g. Bias, assumptions, reasons and interests in the research topic.
*The interviewers are social scientists with many years' experience conducting qualitative research on a range of health topics. This research topic was proposed by Academic Clinical collaborators, and not selected by the interviewers due to any particular prior interests or goals related to this research topic.*

**Domain 2: study design**
*Theoretical framework*
9. Methodological orientation and Theory. What methodological orientation was stated to underpin the study? e.g. grounded theory, discourse analysis, ethnography, phenomenology, content analysis
*In the Methods section we explain how we used semi-structured interviews using thematic analysis with NVivo11 software, this was informed by grounded theory and the constant comparison method.*

*Participant selection*
10. Sampling. How were participants selected? e.g. purposive, convenience, consecutive, snowball.
*Purposive sampling, when clinically convenient, was used to select interviewees in order to attempt to capture maximum variation in views and experiences in order that they adequately reflect those of a range of staff involved in the development, implementation and on-going work in the Integrated Respiratory Service.*

11. Method of approach. How were participants approached? e.g. face-to-face, telephone, mail, email
*Please see item 7 above; moreover, potential participants responded to the initial information from the service leads in both organisations by contacting the researchers directly to discuss the project and/or arrange an interview date and time. This took place at or near the participants' places of work for their convenience. All interviewees gave written informed consent.*

12. Sample size. How many participants were in the study?
*We interviewed 19 members of staff, 8 from the secondary care hospital, 9 from the community provider and 2 who were members of the clinical commissioning group.*

13. Non-participation. How many people refused to participate or dropped out? Reasons
*Only one potential participant was eventually not interviewed. Whilst she was willing, after 3 attempts to meet, clinical duties and shift patterns eventually prevented this. This took place towards the end of the study when data saturation was occurring but other interviews*

*were still taking place. For this reason, as well as to avoid inappropriate pressure on the participant the interview did not take place.*

14. Setting of data collection. Where was the data collected? e.g. home, clinic, workplace
*Data collection took place at or near the participants' place of work, at their preference and convenience.*

15. Presence of non-participants. Was anyone else present besides the participants and researchers?
*These were one to one face to face interviews held in private or in quiet public places, (local café in the community and staff social area which was large enough to ensure privacy and where the participants felt comfortable to express their opinions).*

16. Description of sample. What are the important characteristics of the sample? e.g. demographic data, date.
*All the staff interviewed were involved in the development, implementation and on-going operation of the Integrated Respiratory Service. Interview participants were purposively sampled and recruited to cover the range of staff who worked with the service. (See table 1 in paper)*

*Data collection*
17. Interview guide. Were questions, prompts, guides provided by the authors? Was it pilot tested?
*A topic guide was used for all interviews (provided as an additional file). The focus of the topic guide was participants' views and experiences of the Integrated Respiratory Service and was modified as data collection progressed. The topic guide was used flexibly so that interviewees could raise other issues they felt were important whilst also allowing in-depth exploration of emergent themes.*

18. Repeat interviews.
*We did not conduct repeat interviews*

19. Audio/visual recording. Did the research use audio or visual recording to collect the data?
*Yes, all interviews were audio recorded, with the informed consent of participants.*

20. Field notes. Were field notes made during and/or after the interview or focus group?
*Field notes were not made during this study. Reflexive notes were made on password protected computers to aid the iterative development of the topic guide.*

21. Duration. What was the duration of the interviews or focus group?
*Between 14 and 86 minutes*

22. Data saturation. Was data saturation discussed?
*Yes, this was discussed in team meetings.*

For peer review only

23. Transcripts returned. Were transcripts returned to participants for comment and/or correction?
*No.*

**Domain 3: analysis and findings**
*Data analysis*
24. Number of data coders. How many data coders coded the data?
*Two. Data was coded by the researcher Tracey Stone, and Jon Banks, a senior qualitative research fellow. The coding frame was developed by JB and TS following the initial double coding of four interviews covering a range of staff. Coding and iterative developments were discussed at regular meetings.*

25. Description of the coding tree. Did authors provide a description of the coding tree?
*No.*

26. Derivation of themes. Were themes identified in advance or derived from the data?
*Themes were derived deductively from our research questions and inductively from the data.*

27. Software. What software, if applicable, was used to manage the data?
*We used NVivo 11 qualitative software package to manage the data.*

28. Participant checking. Did participants provide feedback on the findings?
*No.*

29. Quotations presented. Were participant quotations presented to illustrate the themes / findings? Was each quotation identified? e.g. participant number
*Yes, with quotations identified using alphanumeric labels for the site, staff role and interview number. These categories are intentionally quite broad. Whilst readers may appreciate more specific information for context, the sample size and variation of roles within the sample in both organisations, would undermine anonymity.*

30. Data and findings consistent. Was there consistency between the data presented and the findings?
*Yes.*

31. Clarity of major themes. Were major themes clearly presented in the findings?
*Yes.*

32. Clarity of minor themes. Is there a description of diverse cases or discussion of minor themes?
*Yes.*

For peer review only
