# Supplementary material for: Integrating care between an NHS hospital, a community provider and the role of commissioning: the experience of developing an integrated respiratory service
Source: BMJ Open. 2020 Dec 21;10(12):e040267. doi: 10.1136/bmjopen-2020-040267 (PMC7754656; doi:10.1136/bmjopen-2020-040267)
Supplement: Reviewer comments [file bmjopen-2020-040267.reviewer_comments.pdf]

PEER REVIEW HISTORY

BMJ Open publishes all reviews undertaken for accepted manuscripts. Reviewers are asked to complete a checklist review form (**http://bmjopen.bmj.com/site/about/resources/checklist.pdf**) and are provided with free text boxes to elaborate on their assessment. These free text comments are reproduced below.

ARTICLE DETAILS

**TITLE (PROVISIONAL)**	Integrating care between an NHS hospital, a community provider and the role of commissioning: the experience of developing an integrated respiratory service
**AUTHORS**	Banks, Jonathan; Stone, Tracey; Dodd, James

VERSION 1 – REVIEW

**REVIEWER**	Andrew Wilson<br>Norwich Medical School,UK
**REVIEW RETURNED**	15-Jun-2020

**GENERAL COMMENTS**	This is a qualitative assessment of an integrated respiratory service which seems to have had problems with set up and has struggled.<br>I would have been helpful to have the information in the COREC statement contained within the manuscript.<br>Introduction<br>Pg 3 line 53 I suggest giving the name of the region or some details about it<br>Pg 3 line 59. This reads as if the remit was to ensure more care is delivered in the community and advice given by the hospital (pg4 line 24). This seems contrary to the definition of integrated care (pg 1 line 32) which implies a more two-way process.<br>Pg 3 line 37 – the last paragraph should highlight the research gaps in greater detail and there should be a paragraph describing the aims.<br>Patient involvement<br>Pg 6 line 4 – It would have been useful to gain a patient perspective – in fact this might have been quite revealing. I suggest this is a weakness of this study.<br>Methods<br>Please provide the geographical location of this study (unless described in the background)<br>Please provide the start and finish date of the study in the methods section<br>Was the study purposive or convenience sampling? When were the characteristics required for the sampling capture?<br>How experienced was the interviewer?<br>Who did the transcription?<br>Who were the “allied organisational staff”? Why were there no administrative/secretarial staff interviewed from the hospital?<br>Pg 10 Line 38 – this is a description of the IRS and not the research findings so would be better in the background.<br>Pg 12 line 10 – Were the informal communications due to the IRS or independent of it?<br>Discussion<br>Pg 15 line 46 – give the time point

	Pg 17 line 9 contradicts pg 6 line 4<br>I would have liked to see a better discussion of the findings with reference to the existing literature describing studies that have evaluated integrated respiratory services<br>I think there could be better summary and recommendations in the concluding paragraph of the discussion.

**REVIEWER**	Tim Stokes<br>Department of General Practice & Rural Health<br>Otago Medical School<br>University of Otago, Dunedin, NZ
**REVIEW RETURNED**	30-Jun-2020

**GENERAL COMMENTS**	Integrated Care is a mantra for many national health systems in spite of limited evidence for it improving health outcomes. This study is an important and timely addition to the evidence base around delivering integrated respiratory services across primary and secondary care. It reports the qualitative component of an evaluation of such a service in one UK Region (I assume further detail is not given to protect the identity of the specific site). It makes a very important concluding statement that "commissioning an integrated service should incorporate dialogue with stakeholders as early as possible and before the contract is finalised to develop a bedrock of trust".<br><br>Overall this study has appropriate aims and methods, the results are presented clearly and the discussion appropriate. The strengths and limitations are also appropriately stated (including fact that the patient experience of care is not presented).<br><br>Specific requested minor revisions:<br>1. Abstract. Participants, P2, LL22-25. State total numbers of participants first, then break down into groups.<br>2. Abstract. Results. It would be better if this follows the findings in the results - so state the two identified themes and report subthemes for each as appropriate.<br>3. Strengths and Limitations, Bullet point 1. Add commissioners as well.<br>4. Discussion. I would encourage the authors to restructure the discussion so it aligns with BMJ guidance (https://bmjopen.bmj.com/pages/authors/ "We also recommend, but do not insist, that the discussion section is no longer than five paragraphs and follows this overall structure (you do not need to use these as subheadings): a statement of the principal findings; strengths and weaknesses of the study; strengths and weaknesses in relation to other studies, discussing important differences in results; the meaning of the study: possible explanations and implications for clinicians and policymakers; and unanswered questions and future research").<br>Specifically, there should be:<br>a) a short summary of findings before the next section on comparison with existing literature.<br>b) consider adding a para on implications for health policy and practice before ending with a short conclusion.

**REVIEWER**	Tanja Fusi-Schmidhauser, MD PhD<br>Department of Internal Medicine, Ospedale Regionale di Lugano, Ente Ospedaliero Cantonale<br>Switzerland

REVIEW RETURNED	26-Jul-2020

GENERAL COMMENTS	I thank the editor in chief for having given me the opportunity to review this paper and I apologize for the delay due to unexpected leave.<br>This qualitative interview study aimed to explore the perceived efficacy of integrated working between organisations from the perspective of staff in a novel integrated respiratory service. Furthemore, the relationship between commissioning and integration of services was also evaluated.<br>The article is well positioned in the local setting and draws from the current international literature on the topic. It clearly states the objectives of the research, the methods adopted and the results. Data collection and analysis are well described. I appreciated the availability of the interview topic guide as a supplementary file. Discussion and conclusions are well balanced and adequately supported by the findings. Referencing is overall adequate and acknowledges previous published work.<br><br>Following there are some minor comments intended to improve the already good quality of the outlined research.<br><br>1) Although aims are well defined and clearly stated, it would be useful to also state the research question.<br>2) An abbreviation list could help to ease the reading flow.<br>3) Where there specific exclusion criteria? If so, they need to be stated<br>4) I would suggest to highlight the subthemes in the results' section, as it would help to better understand each section<br>5) Study limitations are clearly stated at the beginning of the paper. I would suggest to further discuss them in the "discussion" section

VERSION 1 – AUTHOR RESPONSE

Reviewer: 1
Reviewer Name
Andrew Wilson

Institution and Country
Norwich Medical School,UK

This is a qualitative assessment of an integrated respiratory service which seems to have had problems with set up and has struggled.
I would have been helpful to have the information in the COREC statement contained within the manuscript.
RESPONSE: We understand this request and how it could add to the paper but the word limitations make this unfeasible. Much of the information on the COREQ form around design and analysis is included in the paper. The COREQ form will be available alongside the paper as a supplementary file/appendix.

Introduction

Pg 3 line 59. This reads as if the remit was to ensure more care is delivered in the community and advice given by the hospital (pg4 line 24). This seems contrary to the definition of integrated care (pg 1 line 32) which implies a more two-way process.
RESPONSE: Yes, we recognise the inconsistency here and have reworded the rationale to emphasise the shared aspect of providing care in the integrated respiratory pathway.
'The rationale of the development was that care could be shared between secondary care and the community to the benefit of patients, who could receive care and support in the community, and at the same time reduce demand on secondary care'.

Pg 3 line 37 – the last paragraph should highlight the research gaps in greater detail and there should be a paragraph describing the aims.
RESPONSE: We have rewritten the last paragraph of the introduction to provide greater clarity of the problem being addressed (research on integrated care pathways has shown they have limited impact on patient care) and our study aims (interview staff to understand the nature of the working relationships between the organisations and the impact on the pathway of the commissioning process).
New text below,
'Previous research in relation to integrated respiratory care has identified some promising results in terms of integration and admission avoidance but in general the results have been mixed. Despite the policy prioritisation associated with integrated care in general, research and evaluations have not produced clear evidence of effectiveness. Studies have shown how tensions between the professional cultures of different organisations can impede the integration. The commissioning process (the contractual and clinical governance arrangements that underpin collaboration) has also been identified as shaping the working relationships and quality of integration between organisations involved in care pathways. We undertook a qualitative evaluation of the IRS to understand: (a) the extent to which staff within the IRS worked together to provide an integrated care pathway; and (b) the relationship between commissioning and the development of an integrated service.'

Patient involvement
Pg 6 line 4 – It would have been useful to gain a patient perspective – in fact this might have been quite revealing. I suggest this is a weakness of this study.
RESPONSE: We're not clear whether this point is referring to patient involvement in study design, data collection, i.e. fulfilling a Patient and public involvement role or whether it refers to patients as research participants. We recognise that our sub-heading and text is ambiguous and have changed it to clarify how this section refers to patient and public involvement.
In reference to the lack of patient participation in the study this has been highlighted in the strengths and limitations section bullet points at the beginning of the paper (bullet 5). We have also highlighted it as a limitation in the discussion section of the main paper which now has a strengths and limitations sub-section.

Methods
Please provide the geographical location of this study (unless described in the background)
RESPONSE: Because the organisations involved are relatively small we are concerned that naming the region could compromise anonymity. We have added a line in the introduction to clarify this,
'We have not named the region to preserve participant anonymity'

Please provide the start and finish date of the study in the methods section
RESPONSE: We have added the dates to the start of the methods section,
'The study took place between May 2018 and May 2019'

Was the study purposive or convenience sampling? When were the characteristics required for the sampling capture?

RESPONSE: Para 1 of the methods highlights that sampling was purposive and para 2 gives more details about the sampling criteria,
'Sampling sought to capture the range of roles in the IRS including: senior clinical/management staff, team leads, clinical staff and support staff'.

How experienced was the interviewer?
RESPONSE: The interviews were conducted by JB and TS, we have added text to para 3 of the methods to clarify. Both are experienced qualitative researchers (see COREQ form for details).
'Interviews were conducted by JB and TS and were audio-recorded'

Who did the transcription?
RESPONSE: A commercial transcribing company did the transcribing, we have added this detail in para 4 of the methods,
'Audio recordings of interviews were transcribed by a professional transcribing service who entered into a confidentiality agreement with the research team.'

Who were the "allied organisational staff"? Why were there no administrative/secretarial staff interviewed from the hospital?
RESPONSE: We can see that the term 'allied organisational staff' is confusing. These are the equivalent of health care assistants who supported the clinical nurses and physiotherapists in the community organisation. The equivalent role did not feature in the hospital team in relation to our inclusion criteria, 'staff who were involved in the formation and delivery of the IRS.' We have renamed this group as nursing support staff.

Pg 10 Line 38 – this is a description of the IRS and not the research findings so would be better in the background.
RESPONSE: Yes, we agree and have moved this text to the introduction and appended it to the description of the IRS pathway. We have added text to introduce this section which reads,
'There were differing perspectives on the joint staff meetings …'

Pg 12 line 10 – Were the informal communications due to the IRS or independent of it?
RESPONSE: The informal development of working relationships came about partly because of the IRS, it was the creation of the pathway that brought them together but it was the personal contact forged between practitioners over time rather than the formalised mechanisms that we refer to in the previous section that facilitated effective communication. We have added text to refer to the informal contacts,
'It was these contacts rather than the more formalised group meetings that enabled a more effective and comfortable level of communication.'

Discussion
Pg 15 line 46 – give the time point
RESPONSE: We agree that the timing of the interviews in relation to the timeline of the IRS is relevant but we thought that it would sit better with para one of the results and gives context to the discussion. See below for new additional text,
'Nineteen interviews were conducted between October and December 2018, approximately 2 years after the IRS started.'

Pg 17 line 9 contradicts pg 6 line 4
RESPONSE: We can see how this is confusing but the changes we have made in relation to patient involvement above should clarify this issue, particularly changing the section heading to patient and public involvement.

I would have liked to see a better discussion of the findings with reference to the existing literature describing studies that have evaluated integrated respiratory services
I think there could be better summary and recommendations in the concluding paragraph of the discussion.
RESPONSE: We have added papers to the literature section that relate to integrated respiratory services (refs 21, 22) but these have a different focus to our study. We have also run searches on PubMed and Google Scholar to check for relevant publications as well as the publication repository on respiratory futures a website set up to support the development of integrated respiratory care. It is our argument that our study sits within the more general literature on integrated care where there is an established literature relating to organisational integration and commissioning.
We have organised our discussion so that the comparison with existing literature is marked out more distinctly. For brevity we have not pasted the new para below but each section of the discussion is now marked out with the following subsections,
- Summary
- Comparison with other literature
- Strengths and limitations
- Implications for policy

Reviewer: 2
Reviewer Name
Tim Stokes

Institution and Country
Department of General Practice & Rural Health
Otago Medical School
University of Otago, Dunedin, NZ

Please state any competing interests or state ‘None declared’:
None declared

Please leave your comments for the authors below
Integrated Care is a mantra for many national health systems in spite of limited evidence for it improving health outcomes. This study is an important and timely addition to the evidence base around delivering integrated respiratory services across primary and secondary care. It reports the qualitative component of an evaluation of such a service in one UK Region (I assume further detail is not given to protect the identity of the specific site). It makes a very important concluding statement that "commissioning an integrated service should incorporate dialogue with stakeholders as early as possible and before the contract is finalised to develop a bedrock of trust".
RESPONSE: Thanks for the feedback. We can confirm that details of the region have not been included to maintain anonymity and we have added a line in para 3 of the introduction to make this clear,
‘We have not named the region to preserve participant anonymity.’

Overall this study has appropriate aims and methods, the results are presented clearly and the discussion appropriate. The strengths and limitations are also appropriately stated (including fact that the patient experience of care is not presented).
RESPONSE: Thanks, we have made some edits to the introduction and aims in response to reviewer 1.

Specific requested minor revisions:
1. Abstract. Participants, P2, LL22-25. State total numbers of participants first, then break down into groups.

RESPONSE: We have amended the abstract, new text below,
Nineteen interview participants: nine from the community provider; eight from the hospital; and two from the clinical commissioning group.

2. Abstract. Results. It would be better if this follows the findings in the results - so state the two identified themes and report subthemes for each as appropriate.
RESPONSE: We have rewritten the results and conclusion sections of the abstract which reflects the results from the main paper more accurately. See below,
'Staff identified lack of integration between the organisations characterized by: poor communication, lack of trust, absence of shared information technology, and ineffective integrative initiatives. The commissioning process created barriers to integration including: contractual limitations which prevented pathway development, absence of agreed clinical governance arrangements, and lack of recognition of community work undertaken by hospital staff. Positive working relationships were established over time as staff recognised the skills that each had to offer.'
We have made minor changes to the conclusion to make it distinct from the results
'The commissioning process underpinned the relationship between the organisations and contributed to negative perceptions of the 'other'. Commissioning an integrated service should incorporate dialogue with stakeholders as early as possible and before the contract is finalised to develop a bedrock of trust.'

3. Strengths and Limitations, Bullet point 1. Add commissioners as well.
RESPONSE: We agree and have added commissioners to bullet point 1 of strengths and limitations

4. Discussion. I would encourage the authors to restructure the discussion so it aligns with BMJ guidance (https://bmjopen.bmj.com/pages/authors/ "We also recommend, but do not insist, that the discussion section is no longer than five paragraphs and follows this overall structure (you do not need to use these as subheadings): a statement of the principal findings; strengths and weaknesses of the study; strengths and weaknesses in relation to other studies, discussing important differences in results; the meaning of the study: possible explanations and implications for clinicians and policymakers; and unanswered questions and future research").
Specifically, there should be:
a) a short summary of findings before the next section on comparison with existing literature.
b) consider adding a para on implications for health policy and practice before ending with a short conclusion.
RESPONSE: We recognise that our discussion section lacked structure and we have reorganised and rewritten parts of it which now has the following structure,
- Summary
- Comparison with other literature
- Strengths and limitations
- Implications for policy

Reviewer: 3
Reviewer Name
Tanja Fusi-Schmidhauser, MD PhD

Institution and Country
Department of Internal Medicine, Ospedale Regionale di Lugano, Ente Ospedaliero Cantonale Switzerland

Please state any competing interests or state 'None declared':
None declared

Please leave your comments for the authors below
I thank the editor in chief for having given me the opportunity to review this paper and I apologize for the delay due to unexpected leave.
This qualitative interview study aimed to explore the perceived efficacy of integrated working between organisations from the perspective of staff in a novel integrated respiratory service. Furthemore, the relationship between commissioning and integration of services was also evaluated.
The article is well positioned in the local setting and draws from the current international literature on the topic. It clearly states the objectives of the research, the methods adopted and the results. Data collection and analysis are well described. I appreciated the availability of the interview topic guide as a supplementary file.
Discussion and conclusions are well balanced and adequately supported by the findings. Referencing is overall adequate and acknowledges previous published work.
RESPONSE: Thanks for the feedback

Following there are some minor comments intended to improve the already good quality of the outlined research.

1) Although aims are well defined and clearly stated, it would be useful to also state the research question.
RESPONSE: We have rewritten the aims at the end of the introduction section. The two aims together constitute our research questions.

2) An abbreviation list could help to ease the reading flow.
RESPONSE: We have added a list of abbreviations to the paper just after the abstract. We have also expanded the full title of CSU which is used in the final data extract in the paper (also included in the list below)
CCG: Clinical Commissioning Group
CSU: Commissioning Support Unit (organisations which support the CCGs in commissioning services)
COPD: Chronic obstructive pulmonary disease
COREC Guidelines: Consolidated criteria for reporting qualitative research
HOT: Hospital Outpatient Treatment clinic
ICPs: Integrated care pathways
IRS: Integrated Respiratory Service

3) Where there specific exclusion criteria? If so, they need to be stated
RESPONSE: There were no specified exclusion criteria. Our inclusion criteria have been added to para 1 of the methods,
'Inclusion criteria were staff who were involved in the formation and delivery of the IRS.'

4) I would suggest to highlight the subthemes in the results' section, as it would help to better understand each section
RESPONSE:
We have marked out the two main themes in the results section in 14pt text and the sub themes are now identified with 12pt italicised text. The new section headings in the discussion use 14pt text.

5) Study limitations are clearly stated at the beginning of the paper. I would suggest to further discuss them in the "discussion" section
RESPONSE: We have added more detail and a separate section for strengths and limitations as part of the discussion.

VERSION 2 – REVIEW

**REVIEWER**	Professor Tim Stokes<br>University of Otago<br>Dunedin<br>New Zealand
**REVIEW RETURNED**	04-Sep-2020

**GENERAL COMMENTS**	The authors have provided a detailed and appropriate response to the reviewers. They have appropriately re-worded the abstract, and full paper (in particular the discussion). I consider they have fully addresses the reviewers' concerns.

**REVIEWER**	Tanja Fusi-Schmidhauser<br>Department of Internal Medicine, Ospedale Regionale di Lugano, Switzerland
**REVIEW RETURNED**	04-Oct-2020

**GENERAL COMMENTS**	This paper is now ready for publication.
